# Differentially Abundant Bacterial Taxa Associated with Prognostic Variables of Crohn’s Disease: Results from the IMPACT Study

**DOI:** 10.3390/jcm9061748

**Published:** 2020-06-05

**Authors:** Soo-kyung Park, Han-Na Kim, Chang Hwan Choi, Jong Pil Im, Jae Myung Cha, Chang Soo Eun, Tae-Oh Kim, Sang-Bum Kang, Ki Bae Bang, Hyun Gun Kim, Yunho Jung, Hyuk Yoon, Dong-Soo Han, Chil-Woo Lee, Kwangsung Ahn, Hyung-Lae Kim, Dong Il Park

**Affiliations:** 1 Division of Gastroenterology, Department of Internal Medicine, Kangbuk Samsung Hospital, Sungkyunkwan University School of Medicine, Seoul 03181, Korea; skparkmd@gmail.com; 2 Medical Research Institute, Kangbuk Samsung Hospital, Sungkyunkwan University School of Medicine, Seoul 03181, Korea; hanna147942@gmail.com (H.-N.K.); chilwoo.lee@samsung.com (C.-W.L.); 3 Inflammatory Bowel Disease Study Group of the Korean Association for the Study of Intestinal Diseases, Seoul 06193, Korea; gicch@cau.ac.kr (C.H.C.); jpim0911@snu.ac.kr (J.P.I.); kto0440@paik.ac.kr (T.-O.K.); bodnsoul@hanmail.net (H.Y.); 4 Department of Clinical Research Design and Evaluation, SAIHST, Sungkyunkwan University, Seoul 03063, Korea; 5 Department of Internal Medicine, Chung-Ang University College of Medicine, Seoul 06973, Korea; 6 Department of Internal Medicine and Liver Research Institute, Seoul National University College of Medicine, Seoul 03080, Korea; 7 Department of Internal Medicine, Kyung Hee University Hospital at Gang Dong, Kyung Hee University School of Medicine, Seoul 02447, Korea; clicknox@hanmail.net; 8 Department of Internal Medicine, Hanyang University Guri Hospital, Guri 11923, Korea; cseun@hanyang.ac.kr (C.S.E.); hands@hanyang.ac.kr (D.-S.H.); 9 Division of Gastroenterology, Department of Internal Medicine, Inje University Haeundae Paik Hospital, Busan 48108, Korea; 10 Division of Gastroenterology, Department of Internal medicine, Daejeon St. Mary’s Hospital, The Catholic University of Korea, Daejeon 34943, Korea; sangucsd@gmail.com; 11 Department of Internal Medicine, Dankook University College of Medicine, Cheonan 31116, Korea; kibaebang@gmail.com; 12 Division of Gastroenterology, Department of Internal Medicine, Soonchunhyang University College of Medicine, Seoul Hospital, Seoul 04401, Korea; medgun@schmc.ac.kr; 13 Division of Gastroenterology, Department of Internal Medicine, Soonchunhyang University College of Medicine, Cheonan Hospital, Cheonan 31151, Korea; yoonho7575@naver.com; 14 Department of Internal Medicine, Seoul National University Bundang Hospital, Seongnam 13620, Korea; 15 Functional Genome Institute, PDXen Biosystems Inc., Daejeon 34129, Korea; kwangsung.ahn@gmail.com; 16 Department of Biochemistry, School of Medicine, Ewha Womans University, Seoul 07804, Korea; hyung@ewha.ac.kr

**Keywords:** Crohn’s disease, prognosis, microbiota

## Abstract

Limited studies have examined the intestinal microbiota composition in relation to Crohn’s disease (CD) prognosis. We analyzed the differences in microbial communities and relevant metabolic pathways associated with prognostic variables in patients with CD. We applied 16S rRNA gene sequencing to analyze a cohort of 1110 CD and healthy control (HC) fecal samples. We categorized patients with CD into good (CD-G), intermediate (CD-I) and poor (CD-P) prognosis groups, according to the history of using biologics and intestinal resection. Microbiota α-diversity decreased more in CD-P than CD-G and CD-I. Microbiota ß-diversity in CD-P differed from that in CD-G and CD-I. Thirteen genera and 10 species showed differential abundance between CD-G and CD-P groups. *Escherichia coli* (*p* = 0.001) and species *Producta* (*p* = 0.01) and genera *Lactobacillus* (*p* = 0.003) and Coprococcus (*p* = 0.01) consistently showed differences between CD-G and CD-P groups after adjusting for confounding variables. Functional profiling suggested that the microbial catabolic pathways and pathways related to enterobacterial common antigen and lipopolysaccharide biosynthesis were better represented in the CD-P group than in the CD-G group, and *E. coli* were the top contributors to these pathways. CD prognosis is associated with altered microbiota composition and decreased diversity, and *E. coli* might be causally involved in CD progression, and may have adapted to live in inflammatory environments.

## 1. Introduction

Inflammatory bowel disease (IBD), the most prevalent forms of which are Crohn’s disease (CD) and ulcerative colitis, is a chronic inflammatory gastrointestinal disorder. The pathogenesis of IBD involves a complex interplay among a genetically susceptible host, enteric microbiota, environmental factors, and dysregulated immune response [1,2]. As one such factor, the gut microbiota is gaining increasing attention, owing to its influence on IBD. IBD has emerged as one of the most studied diseases linked to the gut microbiota [3].

Using fecal and mucosal samples and culture-independent techniques, several studies have consistently reported that patients with IBD, in particular patients with CD, are associated with a lower microbial α-diversity and are enriched in several groups of bacteria when compared to the microbial diversity of healthy controls (HC) [4]. Although no single causative microbe has been identified, previous studies have reported the potential contribution of microbial pathogens, such as *Clostridium difficile* [5], enterotoxigenic *Bacteroides fragilis* [6] and *Escherichia coli* [7] to CD disease pathogenesis.

CD has a wide range of clinical symptoms and manifestations with varying phenotypes, and the natural history of CD has shown rates of disease complications ranging from 48% to 52%, five years after diagnosis [8]. Thus, many patients with CD require intensive treatment, such as using various biologics or multiple surgical intervention, and efforts have been undertaken to identify risk factors associated with disease complication. In the past, clinical factors such as disease location at the ileum, perianal disease, early age of diagnosis, smoking, requirement for steroids at first flare, and deep mucosal ulcerations were identified as important risk factors for the development of CD complications [9]. In addition to these clinical factors, a substantial heterogeneity in the disease course of CD suggests the possible involvement of a strong host biological component like the microbiome; there are emerging fields of research focusing on the microbiome, metagenomics and metaproteomics [10].

In this study, we assessed whether there are differences in taxonomic and metabolic microbial signatures among patients with CD with a different clinical course.

## 2. Materials and Methods

### 2.1. Study Subject

The present study was undertaken in parallel with a prospective multicenter study performed by the IMPACT (Identification of the mechanism of the occurrence and progression of Crohn’s disease through integrated analysis on both genetic and environmental factors) study. In 2017, the IMPACT study team was established in Korea and obtained a national grant to organize a prospective CD patient cohort (aged > 8 years), for identification of the mechanism of the occurrence and progression of Crohn’s disease. A total of 16 university hospitals are now participating in this study, and collect clinical data of patients with CD who were newly diagnosed or followed-up in the institutions and biological specimens (including blood, stool and tissue specimens).

To identify the characteristics of the microbiome according to the prognosis of CD, a total of 388 patients with CD who provided stool samples at 14 centers between May 2017 and November 2018 were included in this study. The stool samples were collected on the day of enrollment and if patients were taking antibiotics or probiotics at the time, stool samples were collected after more than 3 months after discontinuation of their medication. Of these, 18 patients were identified to have good prognosis, but since the disease duration was less than 3 years, they were subsequently considered not enough to be categorized as having good prognosis, and were hence excluded. Thus, a total of 370 patients with CD were included in the final analysis.

**Figure 1:**
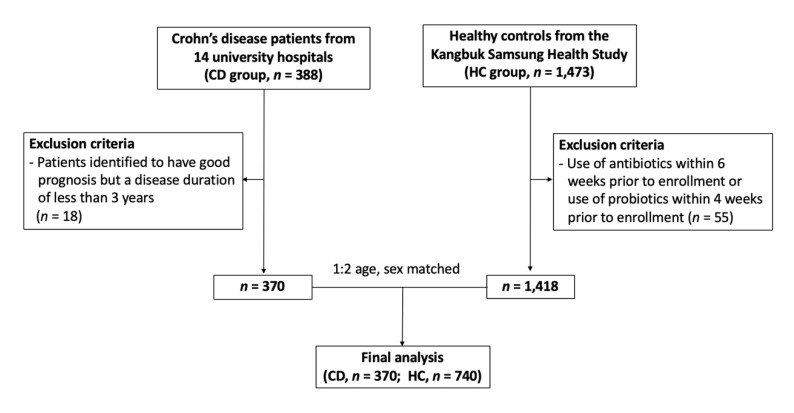
Enrollment of subjects.

For the healthy control (HC) group, we used the fecal microbiome data from the Kangbuk Samsung Health Study, a cohort study of Korean men and women who undergo comprehensive annual or biennial examinations at the Kangbuk Samsung Hospital Healthcare Screening Center. Among the cohort, 1473 adults agreed to provide stool samples between June 2014 and September 2014, and details were described in previous studies [11]. The stool samples were obtained at the day of comprehensive examination, and participants who use antibiotics within 6 weeks prior to enrollment or probiotics within 4 weeks prior to enrollment (*n* = 55) were excluded. These HC were matched for sex and age (±5 years), at a matching ratio of 1:2 with CD group, and in total, 370 patients with CD and 740 HC were included (Figure 1).

Ethical approval of the present study was provided by the institutional review boards of Kangbuk Samsung Hospital (KBSMC 2016-07-029) and each center. Written consent was obtained from all participants after the nature and possible consequences of the studies were explained. All applicable institutional and governmental regulations concerning the ethical use of human volunteers were followed during this research. The research was carried out in accordance with the Declaration of Helsinki.

### 2.2. Data Collection and Group Definitions

For patients with CD, data on diagnosis date, disease location, disease behavior, history of medication, history of surgery and disease activity at fecal sampling were collected through a review of electronic medical report at each center.

Hospitalization and surgery are well known poor clinical outcome in CD. However, we did not consider hospitalization, as indications for CD related hospitalization might be different among centers and physicians. Instead, we considered using biologics as poor outcome as biologics are covered by National Health Insurance Service of Korea only in moderate–severe active Crohn’s disease, which does not respond to treatment with corticosteroids, immunomodulators, or, have no tolerability, or are contraindicated in these treatments. Thus, we categorized these into three prognosis groups as follows: CD-good prognosis group (CD-G): patients who were using or had a history of using only 5-ASA or immunomodulator (azathioprine or 6-MP); CD-intermediate prognosis group (CD-I): patients who were using or had history of using one biologics or had history of one CD-related intestinal resection; CD-poor prognosis group (CD-P): patients who had a history of using one or more biologics with one or more CD-related intestinal resection, had history of using two or more biologics or two or more intestinal resection. Despite the national insurance coverage on the use of biologics in Korea being within those who do not response to corticosteroids or immunomodulators, there is a tendency to use biologics at an earlier state, as it is well known to improve long-term prognosis on patients with CD. In our study, more stringent criteria for our CD-poor prognosis group were devised, which included: patients on whom the first biologic failed and a second (or additional) biologic agent was introduced or required bowel resection; and patients whom, after an initial bowel resection, the worsening of the disease required them to start biologics or undergo a second (or additional) bowel resection.

### 2.3. DNA Extraction and Bacterial 16S rRNA Gene Sequencing

In both CD and HC groups, fecal samples were collected from participants’ homes by self-sampling within 24 hours, before visiting the hospital and immediately frozen at −20 °C freezer after defecation, as instructed. After collection, the samples were stored in a deep-freezer (−70 °C) in the laboratory, as soon as the participants arrived at the hospital. Details including DNA extraction from stool samples and 16S rRNA gene sequencing in HC were described in previous studies [11]. 

In patients with CD, DNA from stool samples was extracted within one month after storage using the Stool DNA Isolation Kit (#27600, NIRGEN BIOTEK CORP, Thorold, ON, Canada), according to the manufacturer’s instructions. 

The V3‒V4 region of the 16S rRNA gene was amplified using the 341F and 805R primers, with added Illumina adaptor overhang sequences, 341F (5′ TCG TCG GCA GCG TCA GAT GTG TAT AAG AGA CAG CCT ACG GGN GGC WGC AG 3′) and 805R (5′ GTC TCG TGG GCT CGG AGA TGT GTA TAA GAG ACA GGA CTA CHV GGG TAT CTA ATC C 3′). Amplicons were purified with a magnetic bead-based clean-up system (Agencourt AMPure XP, Beckman Coulter, Brea, CA, USA). Indexed libraries were prepared by limited-cycle PCR using Nextera technology, further cleaned up, and pooled at equimolar concentrations. The final library was denatured with 0.2 N NaOH and diluted to 6 pM with a 20% PhiX control. Sequencing and demultiplexing were performed on Illumina MiSeq platform using a 2 × 300 bp paired-end protocol, according to the manufacturer’s instructions. DADA2 plugin of the QIIME2 package (version 2018.11, https://qiime2.org) [12] was used to performed the sequence quality control, such as filtering low quality sequences and chimera, and to construct the feature table of amplicon sequence variants (ASVs). The ASVs were generated by denoising with DADA2 and regarded as 100% operational taxonomic units (OTUs). For taxonomic structure analysis, taxonomy was assigned to ASVs using a pre-trained Naïve Bayes classifier and the q2-feature-classifier plugin against the Greengene 13_8 99% OTUs of the 16S rRNA gene sequence database.

### 2.4. Statistical Analyses

Analyses for baseline clinical characteristics were performed using SPSS software (SPSS 24.0 for Windows; SPSS, Chicago, IL, USA).

#### 2.4.1. Alpha and Beta Diversity

For diversity analysis, the feature table was rarefied to 10,000 sequences per sample by random subsampling in QIIME2. To evaluate the alpha diversity, we computed the number of ASVs observed in each sample, Shannon index accounting both evenness and richness, and Faith’s phylogenetic diversity (PD) [13]. The Kruskal–Wallis test as a non-parametric statistical test was used to test difference for all or pairwise groups. For measuring beta diversity, we used UniFrac distance [14] to estimate dissimilarity among group membership, by incorporating the phylogenetic distances between ASVs. The unweighted and weighted UniFrac distance were calculated for the presence/absence and the abundance of ASVs, respectively, and permutational multivariate analysis of variance (PERMANOVA) with 999 random permutations was used to test significance among groups. PERMANOVA pairwise comparisons for post-hoc test were then conducted to test for significant differences between two groups using the Benjamini and Hochberg (FDR) correction (*q*-value < 0.05).

#### 2.4.2. Microbial Taxonomic Composition between Groups

An analysis of the composition of microbiome (ANCOM) [15] test was performed to determine if there were significant differences in the relative abundance of any taxa between two groups (HC vs. CD and CD-G vs. CD-P), across multiple taxonomic levels. ANCOM accounts for the compositional nature of the relative abundance of taxa. Linear discriminant analysis effect size (LEfSe) analysis was used to detect potential CD progression-specific bacterial markers [16]. Finally, to adjust for confounding factors (age, location, behavior, disease activity and bowel resection history), we applied multivariate association with linear models (MaAsLin) [17], which has the capability to deal with covariates, and compared the abundance of taxa between the CD-G and CD-P group.

#### 2.4.3. Microbial Functional Profiling and Metabolic Pathways

Functional inferences of the microbial community were conducted according to the Phylogenetic Investigation of Communities by Reconstruction of Unobserved States (PICRUSt). We performed PICRUSt2 (v2.2.0-b) [18] with ASVs, according to the instructions (https://github.com/picrust/picrust2/wiki). Phylogenetic placement in PICRUSt2 is based on a sequence of three steps: HMMER (www.hmmer.org) to place ASVs, EPA-NG to determine the best position of these placed ASVs in a reference phylogeny, and GAPPA to output a tree of the most likely ASV placements. This results in a phylogenetic tree containing both reference genomes and environmentally sampled organisms, which is used to predict individual gene family copy numbers for each ASV. PICRUSt2 predictions based on the following gene families are supported by enzyme classification numbers (EC numbers, as of 21 Jan 2016). We generated PICRUSt2 EC gene family predictions and Metabolic Pathway Database (Metacyc) pathway abundance predictions [19]. Results were visualized in statistical analysis of taxonomic and functional profiles (STAMP) version 2.1.3 [20], and tested using Welch’s *t*-test for two groups, CD-G vs. CD-P. All predictions were adjusted by multiple testing correction (Bonferroni *q*-value < 0.05).

## 3. Results

### 3.1. Demographics of the Patients

The baseline characteristics of patients with CD, according to the prognosis groups, are presented in Table 1. Among the 370 patients, 132 (35.7%) were in the CD-G group, 151 (40.8%) were in the CD-I group and 87 (23.5%) were in the CD-P group. The CD-P group (*n* = 87) included the patients with one biologics + one bowel resection (*n* = 36), one biologics + two bowel resection (*n* = 9), one biologics + three bowel resection (*n* = 1), two biologics (*n* = 16), two biologics + one bowel resection (*n* = 11), two biologics + two bowel resection (*n* = 6), three biologics (*n* = 3), three biologics + one bowel resection (*n* = 3) and four biologics (*n* = 1).

Age at diagnosis was more likely to be lower in the CD-P group, as A1 was 6.0%, 9.3%, and 12.6% in CD-G, CD-I, and CD-P groups, respectively (*p* = 0.003). Most of the patients were in remission state (93.3%, 89.2%, and 85.2% in CD-G, CD-I, and CD-P groups, respectively) during sampling. There were no differences in location between groups, but when we classified location as L1, L2, L3, regardless of the presence of L4, L3 tended to show a higher proportion in the CD-P group (76.1%) than in the CD-G (60.2%) and CD-I groups (57.4%) (*p* = 0.05). In behavior, there was a significantly higher proportion of B3 in CD-P group (6.0%, 14.2% and 28.4% % in CD-G, CD-I and CD-P group, respectively *p* < 0.001), and when we classified behavior into three groups (B1, B2, and B3) regardless of perianal involvement, B3 was increased up to 47.4% in the CD-P group (7.5% and 20.9% in the CD-G and CD-I group, respectively). In CD-P group, the biologics most commonly used by the study participants were Infliximab followed by adalimumab, vedolizimab, and ustekinumab. Other biologics that were used, such as risankizumab (protocol number 1311.6), etrolizumab (protocol number GA29144), mongersen (protocol number GED-0301-CD-002), and PF-00547659 (anti MAdCAM-1 antibody, protocol number A7281007), JNJ-64304500, (NKG2D receptor blocker, protocol number 64304500CRD2001), were in open label clinical trials at the time of this study. Bowel resection history was present in 75% of people in the CD-P group. The mean age of the HC group was 41.7 ± 7.5 and 73.5% of them were male and their mean BMI was 24.1 ± 3.2.

### 3.2. Bacterial Community Structure and Diversity among CD Groups and HC

**Figure 2:**
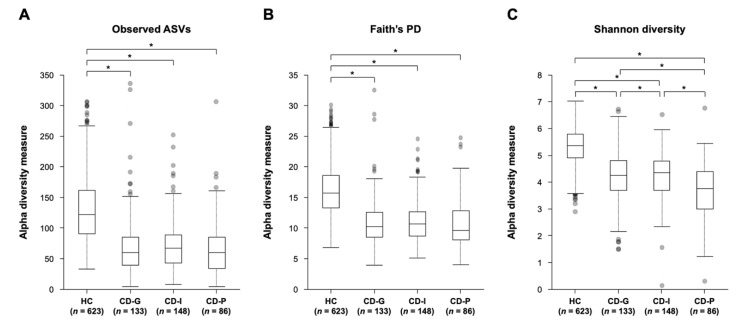
Alpha diversity comparisons of gut microbial communities among groups, according to the Crohn’s disease prognosis. (**A**) Observed ASVs, (**B**) Faith’s phylogenetic diversity, (**C**) Shannon index. * *q* < 0.05 (pairwise Kruskal-Wallis test, Benjamini-Hochberg correction). CD-G = good prognosis group; CD-I = intermediate prognosis group, CD-P = poor prognosis group; HC = health control.

The 16S rRNA gene amplicon sequences after QC ranged from 3865 to 949,266 reads per sample (mean = 45,174) and 14,627 features in 1110 subjects. After rarefying the feature tables to 10,000 sequences per sample, HC (*n* = 623), CD-G (*n* = 133), CD-I (*n* = 148) and CD-P (*n* = 86) were included in the diversity analysis. The alpha diversity of gut microbiota among the HC, CD-G, CD-I, and CD-P groups showed statistically significant differences in the observed ASVs (*p* = 8.5 × 10^−62^), Faith’s PD (*p* = 7.6 × 10^−71^), and Shannon’s index (*p* = 3.4 × 10^−77^). In the pairwise Kruskal–Wallis test, we confirmed significant differences between the HC and CD-G/CD-I/CD-P in observed ASVs, Faith’s PD, and Shannon index (Figure 2). There were significant differences between the Shannon index of the CD-G and CD-P (*q* = 1.5 × 10^−3^) and CD-I and CD-P groups (*q* = 2.4 × 10^−3^). The CD-P group had lower richness than the CD-G and CD-I groups, as shown in Figure 2 and Table S1.

**Figure 3:**
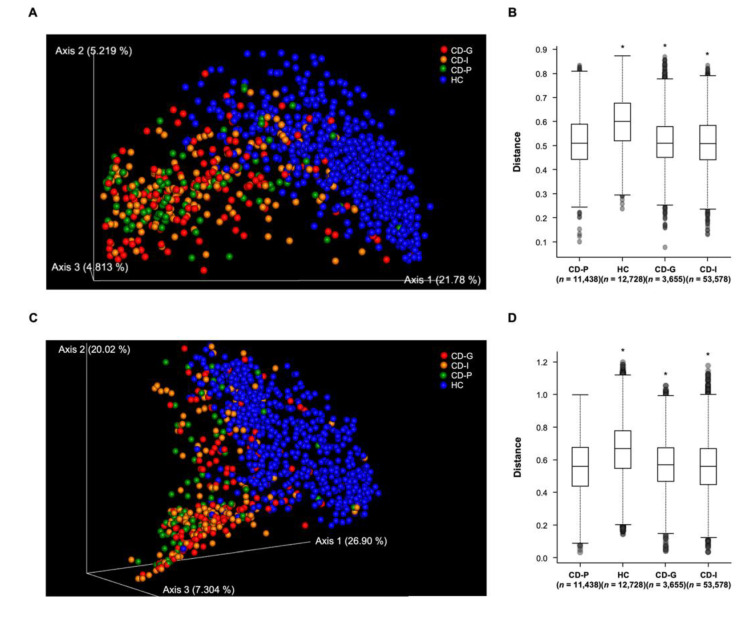
Beta diversity comparisons of gut microbial communities among groups, according to the Crohn’s disease prognosis. (**A**) Unweighted UniFrac distance by 3D principal coordinates analysis (PCoA) plot, (**B**) Unweighted UniFrac distance by boxplot, (**C**) Weighted UniFrac distance by PCoA plot, (**D**) Weighted UniFrac distance by boxplot. The y-axes represent the distance of each group to the CD-P group (baseline). * *q* < 0.05 (pairwise PERMANOVA); CD-G = good prognosis group; CD-I = intermediate prognosis group; CD-P = poor prognosis group; HC = health control.

The phylogenic distance indices in both the weighted- and unweighted-Unifrac distance revealed that microbial communities were significantly different among all four groups (PERMANOVA *p* = 0.001, pseudo-F = 54.91; *p* = 0.001, pseudo-F = 65.96, respectively). In pairwise comparisons, the unweighted Unifrac distance used for identifying the presence/absence of ASVs between groups differed between all pairs of groups, except CD-G and CD-I groups. Weighted Unifrac distance also showed significant differences in the gut microbial community composition between all pairs of groups, except for CD-G and CD-I groups (Table S2 for full pairwise results). However, owing to a high sample number and inter individual variation, the fecal microbiota between the CD-G and CD-P groups could not be separated clearly by principal coordinates analysis (Figure 3A,C), although the differences in microbial community composition were significant between the two groups in both beta diversity indices (Figure 3B,D).

### 3.3. Taxa Associated with Prognosis of CD

To better understand how the microbial community composition changed with the prognosis of CD, we investigated which organisms were present at different taxonomic levels and their relative abundance. Overall, 18 phyla, 39 classes, 71 orders, 129 families, 314 genera, and 459 species were detected. Bacterioidetes (27.6%), Firmicutes (52.7%), Proteobacteria (12.1%), and Actinobacteria (6.6%) were the most abundant phyla across all subjects. Other phyla identified, such as Verrucomicrobia, Fusobacteria, and Tenericutes, had relatively low abundance (<1%).

### 3.4. Comparing the Phylum through Species Levels of Microbial Composition between the HC and CD

First, we compared the phylum through species levels of microbial composition between the HC and CD using ANCOM. The W statistic for the significantly different taxa, relative to more than 90 percent other taxa at each taxon, is shown in Table S3. Differential abundance testing revealed 5 phyla, 11 classes, 9 orders, 22 families, 18 genera, and 12 species that were differentially abundant between HC and CD groups. The phyla Actinobacteria (*w* = 9) and Firmicutes (*w* = 9), including their classes Coriobacteria (*w* = 17), Bacilli (*w* = 14) and Clostridia (*w* = 14), had significantly different abundance across the four groups. The “*w* = 14” of Clostridia indicates that the class was significantly different relative to 14 other classes between the two groups.

### 3.5. Comparing the Phylum through Species Levels of Microbial Composition between the CD-G and CD-P Groups

**Figure 4:**
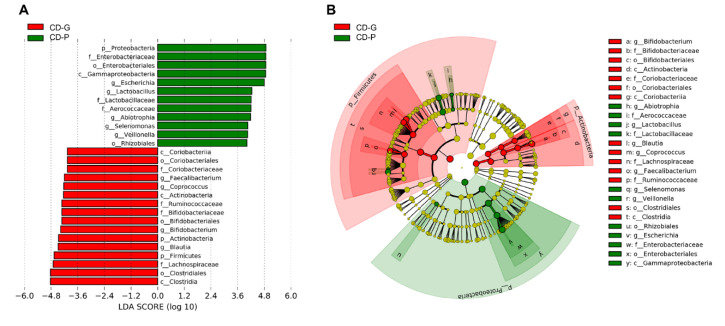
Differentially abundant bacterial taxa between good prognosis (CD-G) and poor prognosis (CD-P) groups in Crohn’s disease. (**A**) A forest plot showing the LDA score indicating significant differences in the bacterial taxa between the CD-G (red) and CD-P (green) groups (LDA score >4.0; *p* < 0.05). (**B**) Cladogram generated using the LEfSe method, indicating the phylogenetic distribution of microbes associated with the CD-G and CD-P groups. CD-G = good prognosis group; CD-P = poor prognosis group; LDA = linear discriminant analysis.

Secondly, we compared the genera and species levels between the CD-G and CD-P groups within the patients with CD. In Table 2, genera and species that had different abundance between the CD-G and CD-P groups, either by the ANCOM analysis (W statistic for the significantly different taxa relative to more than 90 percent other taxa) or LEfSe analysis (with LDA score > 4), are listed. Among the genera and species that had different abundance between HC and CD (Table S1), genera *Enterococcus*, *Lactobacillus*, *Blautia*, *Megasphaera*, *Veillonella*, *Fusobacterium*, *Escherichia*, and *Klebsiella* and the species *V. dispar* and *E. coli* had different abundances between the CD-G and CD-P groups by the ANCOM analysis. LEfSe analysis revealed that CD-G had a significantly higher abundance of genera *Blautia* and *Fusobacterium* than the CD-P group, and CD-P had a significantly higher abundance of genera *Lactobacillus*, *Megasphaera*, *Veillonella*, *Escherichia*, and *Klebsiella* and the species *V. dispar* and *E. coli* than the CD-G. Figure 4 depicts the LEfSe results (LDA score > 4) from phylum to genus level. In addition to the ANCOM result, the genera *Abiotrophia* and *Selenomonas* had a higher abundance in CD-P than the CD-G group and *Faecalibacterium*, *Coprococcus* and *Bifidobacterium* showed higher abundances in CD-G than in the CD-P group.

### 3.6. Significantly Different Taxa between the CD-G and CD-P Groups after Adjusting for Confounding Variables

**Figure 5:**
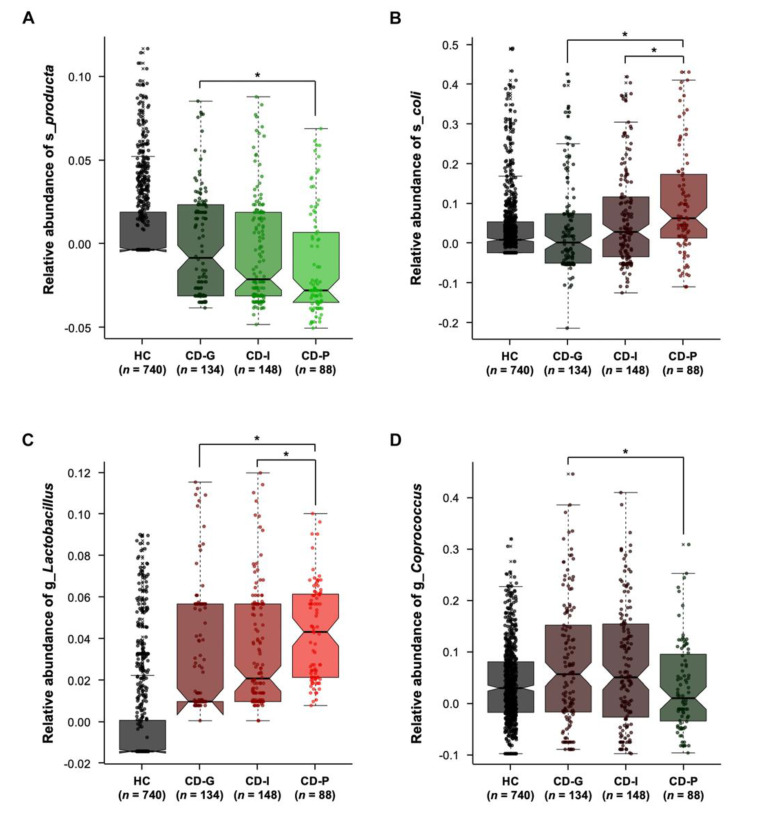
Two species (**A**,**B**) and two genera (**C**,**D**) consistently showed differences between good prognosis and poor prognosis groups after adjusting for confounding variables, when analyzed by the generalized linear models using MaAsLin packages. * *p* < 0.05 CD-G = good prognosis group; CD-I = intermediate prognosis group; CD-P = poor prognosis group; HC = health control.

Thirdly, we used the generalized linear models using MaAsLin packages [17], which have the capability of dealing with covariates, to investigate significantly different taxa between the CD-G and CD-P groups. It is well known that CD prognosis is related to age, CD location, and CD behavior at diagnosis, and these factors showed difference among the CD prognosis group (Table 2). In addition, as stool microbiome might be affected by CD activity at sampling and bowel resection state, we also considered these factors as confounding variables. Among the 13 genera and 10 species that showed different abundance between CD-G and CD-P groups by either ANCOM or LEfSe analysis (Table 2), two species and two genera consistently showed difference after adjusting for confounding variable (Figure 5). An increased level of species *producta* (Figure 5A) and genus *Coprococcus* (Figure 5D) was maintained in the CD-G group, relative to those with CD-P groups (both *p*-value = 0.01). Species *E. coli* (Figure 5B) and genus *Lactobacillus* (Figure 5C) were significantly enriched in the CD-P group than in the CD-G group (*p* = 0.001 and *p* = 0.002, respectively) and in the CD-I group (*p* = 0.03 and *p* = 0.02, respectively).

Among other organisms that had a different abundance between CD-G and CD-P groups, *Faecalibacterium prausnitzii*, a well-known anti-inflammatory organism, that is considered to be a marker of health, showed no difference between CD-G and CD-P groups after adjusting for confounding variables (CD-G and CD-I, *p* = 0.18, CD-G vs. CD-P, *p* = 0.24).

### 3.7. Functional Profiling Related to the CD Prognosis Using PICRUSt2

To understand the gut microbial functions related to the CD prognosis, we used PICRUSt2 to infer putative metagenomes from 16S rRNA gene profiles. STAMP was used to identify microbially relevant functions linked with the CD prognosis. Among the predicted MetaCyc pathways inferred by PICRUSt2 for ASVs, 95 pathways passed the significant thresholds (*q* < 0.05) (Table S4) and 27 pathways among them satisfied the specified filter of effect size (ratio of mean proportions of sequences between groups >2.5) (Figure S1) in CD-G and CD-P. Microbial catabolic pathways such as aromatic compound degradation (4-hydroxyphenylacetate degradation, 3-phenylpropanoate and 3-propanoate degradation to 2-oxopent-4-enoate, superpathway of phenylethylamine degradation, phenylacetate degradation I, etc.), carboxylate degradation (D-glucarate degradation I, D-galactarate degradation I, etc.), and fatty acid degradation (fatty acid beta oxidation I) were present at higher levels in the CD-P than in the CD-G. The family Enterobacteriaceae or its species *E. coli* were the top contributors of the catabolic pathways. Sequences for the metabolism and biosynthesis of ubiquinol (ubiguinol-7, 8, 9, 10 biosynthesis), enterobacteria common antigen (enterobacterial common antigen biosynthesis), heme (superpathway of heme biosynthesis from glycine), lipopolysaccharide (LPS) (superpathway of lipopolysaccharide biosynthesis), and ppGpp (ppGpp biosynthesis) were also increased in the CD-P group. Additionally, enzyme classification numbers (EC numbers) were predicted with PICRUSt2 and 103 EC numbers, including that of flavin reductase (NADH), glyoxylate reductase (NADP^+^), nitrate reductase, and formate dehydrogenase, were significantly different between good (CD-G) and poor groups (CD-P) in CD (all *q*-value < 0.05) (Table S5).

## 4. Discussion

Using a bioinformatics approach and a large multicenter cohort data, this study highlighted the fact that the prognosis of CD is characterized by unique microbial signatures. The CD-P group had lower richness with respect to the α-diversity than the CD-G and CD-I groups. Moreover, ß-diversity indicated significant differences in the composition of the gut microbial community between CD-P vs. CD-G and CD-P vs. CD-I groups. Among the 18 genera and 12 species that were differentially abundant between HC and CD groups, 13 genera and 10 species showed differences in abundance between CD-G and CD-P groups, when analyzed by either ANCOM or LEfSe. *E. coli* from the phylum, Proteobacteria and species *producta*, and *Lactobacillus* and *Coprococcus* genera from the phylum, Firmicutes consistently showed differences between CD-G and CD-P groups after adjusting for confounding variables. Functional profiling using PICRUSt2 suggested that the microbial catabolic pathways, such as aromatic compound degradation, carboxylate degradation, and fatty acid degradation were better represented in the CD-P group than in the CD-G group.

The advance in DNA sequencing technology and analysis have set the stage for investigations into the IBD microbiome. Recent studies consistently report a decrease in diversity along with a decreased representation of several taxa such as Bacteroides, and Clostridia, and an increase in the Gammaproteobacteria, and the presence of *E. coli* or *Fusobacterium* when compared to HC [21]. However, beyond the association between microbiome and IBD pathogenesis, the role of the microbiota at various phases of the disease or for the complicated disease course of CD is less well appreciated. Dovrolis et al. [22] reported that the α-diversity of the microbiota was decreased within all 3 CD behavior phenotypes (B1, B2, and B3) vs. HC, with more significant reductions in B2 and B3 compared with B1. Microbial composition was similar in B2 and B3 samples and it was different from those of B1 and HC. An abundance analysis of microbial families identified significant differences between the B2 and B3 and the B1 phenotype. Solok et al. [23] reported that when compared with a non-recurrence setting, endoscopic recurrence is associated with strong changes in the ileal mucosa-associated microbiota, that are highly reminiscent of those observed generally in ileal CD, with a reduction in the alpha diversity, an increase in the abundance of several members of the phylum, Proteobacteria, and a decrease in the abundance of several members of the Lachnospiraceae and the Ruminococcaceae families within the phylum, Firmicutes.

In our study, we categorized patients with CD into three groups according to their prognosis, and the CD-P group had lower richness than the CD-G and CD-I groups and significant differences in the gut microbial community composition when compared to the CD-G and CD-I groups. Within the Firmicutes phylum, bacteria from the order, Clostridiales and notably those from the Lachnospiraceae families, including *Coprococcus* and *Blautia producta*, have decreased abundance in patients with poor prognosis. In the previous studies, bacteria of the Firmicutes phylum belonging to the Clostridiales order and the Lachnospiraceae families was frequently reported as having decreased abundance in CD compared with that in HC [24], especially in the naïve patients with CD [21]. In addition, as a decreased level of the Lachnospiraceae families was associated with post op recurrence [23], it might be associated with not only pathogenesis, but also the progression of CD. In this study, we could specify the genus (*Coprococcus*) and species (*Blautia producta*) level among the Lachnospiraceae families. However, within the Firmicutes phylum, members of the Bacilli class, such as Lactobacillus genera, increased in patients with poor prognosis. This was in contradiction to our expectation, as Lactobacillus is well known to be a protective bacterium in IBD, by the down regulating of inflammatory cytokines in gut mucosa [25]. The increased levels of *Lactobacillus* were unlikely due to the intake of probiotic, as we enrolled the patients after discontinuing the probiotics, and certain studies have shown an increase in the Lactobacillus in IBD patients [26,27]. As it also increased in B2, and B3 phenotype than B1 [22], the controversies on this bacterium and its role in the pathogenesis of IBD should be further investigated.

It is well known that the relative proportions of Proteobacteria, including *E. coli*, are increased in the mucosa of IBD patients [21,28,29]. Among the *E. coli* strains, adherent invasive *E. coli* (AIEC) has the ability to adhere to and invade intestinal epithelial cells (IEC), as well as to replicate within macrophages [30]. Several molecular and culture-based investigations support a putative role of AIEC in CD [31]. In addition, there are some data supporting the role of *E. coli* infection in regulating the activity and severity of CD. Mylonaki et al. [32] demonstrated an increased number of *E. coli* in the epithelium and lamina propria of patients with active CD in comparison to their number in inactive CD. Elliott et al. [33] found an association between the abundance of mucosa-associated *E. coli* and the severity of endoscopic inflammation, and several studies have associated the presence of AIEC with ileal recurrence after surgery for CD. As *E. coli* was increased in the CD-P group after adjusting these confounding factors, such as disease location, behavior, activity and surgery, the results of the present study corresponded with the results of previous studies and suggested an independent association of *E. coli* with CD prognosis.

Our PICRUSt2 results identify metabolic signatures associated to inflammation-associated dysbiosis and might provide insight about the role of selective growth of *E. coli* in CD. Firstly, the poor prognosis group was associated with increased microbial catabolic pathways, and the family Enterobacteriaceae or its species *E. coli* was the top contributor to the catabolic pathways. Inflammation is a heavily energy-consuming process, as the activation, proliferation and recruitment of cells require energy-enriched substrates involved in inflammation and immunity [34]. Bacteria form a cell-wall containing organisms and require large amounts of carbohydrates during growth for the biosynthesis of complex structural polysaccharide. During inflamed status, available carbohydrates are absent and the glyoxylate cycle permits the synthesis of glucose from lipids via acetate generated in fatty acid β-oxidation. We identified that several compound degradation pathways were over-represented in the CD-P group. These pathways ultimately yield precursor metabolites, which is further degraded to pyruvate and acetyl-CoA to enter into the TCA cycle. *E. coli* is a prototropic facultative anaerobe which can use these compounds as the sole source of carbon for growth, and has the ability to respire oxygen, use alternative anaerobic electron acceptors, or ferment [35]. Thus, one of the potential mechanisms by which *E. coli* survives, despite the inflammatory environment, is their growth and anaerobic respiration by the utilization of stable reactive oxygen and nitrogen produced as by-products of the host inflammatory response [36]. Second, we found that pathways related to enterobacterial common antigen and LPS biosynthesis were highly represented in the CD-P group in comparison to their representation in the CD-G group. High levels of *E. coli* colonization in the gut are correlated with high concentrations of its LPS, which activates the host immune system. LPS is recognized by toll-like-receptor (TLR) 4, and TLRs are upregulated at inflamed tissue sites [37]. Additionally, gram-negative bacteria have several strategies to defend themselves from polymyxin antibiotics (polymyxin B and colistin), including a variety of LPS modifications. The polymyxin resistance pathway was highly detected in the CD-P group. Third, the enterobactin pathway was highly detected in the CD-P group, and a previous study showed that enterobactin, a catecholate siderophore secreted by *E. coli*, inhibits the activity of the neutrophil bactericidal enzyme and myeloperoxidase, both in vitro, hence promoting the survival of *E. coli* in the inflammatory environment [38].

We demonstrated, for the first time, that microbiome associated to the poor prognosis group is different from that associated to the good or intermediate prognosis group in CD by the large multicenter cohort. In addition to the organisms previously implicated in CD, including the Lachnospiraceae and Enterobacteriaceae families, we identified that organisms belonging to the genus *Coprococcus*, and *Blautia* of Lachnospiraceae family and *E. coli* of Enterobacteriaceae family showed difference in abundance between the different CD prognosis groups. Although the analysis dealing with covariates resulted in only a short list of taxa significantly associated with CD prognosis, these taxa might be independently associated with CD prognosis, in addition to clinical factors. In agreement with previous studies on CD cohorts from western countries, the decrease of Lachnospiraceae family and increased of *E. coli* was consistently observed in our study in Korean patients with CD, although NOD2 and ATG16L1 mutations, which are related with bacterial handling, are not frequently observed in Korean patients with CD [39]. These results can be helpful to understand the pathogenesis of CD in different genetic and epidemiologic backgrounds. Finally, our PICRUSt2 results provide insight about the mechanisms of selective growth of *E. coli* in patients with CD, that *E. coli* might not only be causally involved in patients with CD, but may have also merely adapted to live in this affected environment.

This study has several limitations. First, the retrospective, cross-sectional nature of this study precludes definite conclusions as to whether the changes of intestinal microbial composition according to the CD prognosis are primary or secondary events. So far, an informative study of pediatric patients with CDconfirms the association between the disease state, alterations to bacterial taxa and reduction in diversity in treatment-naive CD cohort [21]. Second, we collected stool sample only one time at enrollment. However, bacteria, such as *E. coli*, contributed to a large number of shifts in outcome of IBD [40]. In order to validate these results, future work will be needed to consistently collect samples from patients over a period of extensive disease durations. Third, in a new-onset CD study, microbial balance is less shifted toward a dysbiotic state in the lumen, supporting the need to examine mucosal tissue in addition to stool samples [21]. However, the large-scale collection of stool samples is an even less invasive approach, and some bacteria such as those belonging to the *Escherichia*/*Shigella* genus were predominantly observed in both mucosal tissues and stool samples of patients with CDcompared to that in HC [24]. Large studies involving gut microbiome profiling should incorporate mucosal microbiome sampling in addition to fecal sampling in the future, and should compare their effectiveness as a biomarker.

In conclusion, CD prognosis is associated with changes in the composition of the microbiota and decreased diversity. Further studies of a prospective nature and a longitudinal design are needed to confirm that particular bacteria can be used as a predictor of disease progression.

## Figures and Tables

**Table 1:** Baseline characteristics of the patients with Crohn’s disease (CD) patients.

Baseline Characteristics	CD-G (*n* = 134)	CD-I (*n* = 148)	CD-P (*n* = 88)	*p*-Value
Age at diagnosis ^a^				0.003
A1	8 (6.0)	14 (9.3)	11 (12.6)	
A2	92 (68.7)	116 (78.4)	70 (79.5)	
A3	34 (25.4)	18 (12.2)	7 (8.0)	
Age at sampling	36.9 ± 14.2	32.9 ± 10.1	36.8 ± 10.9	0.008
Sex, Male	99 (73.9)	107 (72.3)	66 (75.0)	0.89
Disease duration	6.2 ± 4.3	6.3 ± 4.9	10.9 ± 6.7	<0.001
BMI, kg/m^2^	23.5 ± 3.4	22.9 ± 21.6	21.6 ± 3.8	<0.001
CDAI ^b^				0.07
Remission	125 (93.3)	132 (89.2)	75 (85.2)	
Mild	6 (4.5)	3 (2.0)	5 (5.7)	
Moderate to Severe	3 (2.2)	13 (8.8)	8 (9.1)	
Location ^a,b^				0.16
L1	34 (25.4)	35 (23.6)	15 (17.0)	
L2	14 (10.4)	18 (12.2)	5 (5.7)	
L3	68 (50.7)	68 (45.9)	57 (64.8)	
L1 + L4	5 (3.7)	8 (5.4)	0	
L2 + L4	0	2 (1.4)	1 (1.1)	
L3 + L4	12 (9.0)	17 (11.5)	10 (11.4)	
L4	1 (0.7)	0	0	
Behavior ^a,b^				<0.001
B1	85 (63.4)	59 (39.9)	13 (14.8)	
B2	9 (6.7)	22 (14.9)	13 (14.8)	
B3	8 (6.0)	21 (14.2)	25 (28.4)	
B1 + P	29 (21.6)	32 (21.6)	13 (14.8)	
B2 + P	1 (0.7)	4 (2.7)	7 (8.0)	
B3 + P	1 (0.7)	10 (6.8)	17 (19.3)	
Medication ^c^				
5-ASA	130 (97.0)	119 (80.4)	61 (69.3)	<0.001
Steroid	58 (43.2)	77 (52.0)	44 (50.0)	0.32
Immunomodulator	105 (78.4)	144 (97.3)	82 (93.2)	<0.001
Biologics	0	129 (87.2)	87 (84.1)	<0.001
IFN	0	106 (71.6)	40 (45.5)	<0.001
ADA	0	23 (15.5)	22 (25.0)	<0.001
Others ^d^	0	0		<0.001
Bowel resection history	0	19 (12.8)	66 (75.0)	<0.001
Small bowel	0	7 (4.7)	29 (33.3)	<0.001
Colon	0	8 (5.4)	21 (23.9)	
Small bowel + colon	0	4 (2.7)	16 (18.2)	

^a^ Age at diagnosis, location and behavior were according to the Montreal classification. ^b^ CDAI, Location and behavior at sampling date. CDAI <150: remission, 150‒220: mild, >221: moderate to severe. ^c^ Medication included both past and current medication. ^d^ Others included vedolizimab, risankizumab, etrolizumab, ustekinumab, mongersen, PF-00547659 (anti MAdCAM-1 antibody) and JNJ-64304500, (NKG2D receptor blocker). ADA = adalimumab; ASA = aminosalicylic acid; BMI = body mass index; CDAI = Crohn’s disease activity index; CD-G = good prognosis group; CD-I = intermediate prognosis group; CD-P = poor prognosis group; IFN = infliximab.

**Table 2:** Significantly different taxa between the CD-G and CD-P groups.

Taxa Level ^a^	Taxonomic Assignment	W ^b^	Group ^c^	LDA Score ^d^
Genus	p_Actinobacteria;c_Actinobacteria;o_Bifidobacteriales;f_Bifidobacteriaceae;g_Bifidobacterium	ns	CD-G	4.4
	p_Firmicutes;c_Bacilli;o_Lactobacillales;f_Aerococcaceae;g_Abiotrophia	ns	CD-P	4.2
	p_Firmicutes;c_Bacilli;o_Lactobacillales;f_Enterococcaceae;g_Enterococcus	53	ns	
	p_Firmicutes;c_Bacilli;o_Lactobacillales;f_Lactobacillaceae;g_Lactobacillus	45	CD-P	4.2
	p_Firmicutes;c_Clostridia;o_Clostridiales;f_Lachnospiraceae;g_Blautia	51	CD-G	4.5
	p_Firmicutes;c_Clostridia;o_Clostridiales;f_Lachnospiraceae;g_Coprococcus	ns	CD-G	4.2
	p_Firmicutes;c_Clostridia;o_Clostridiales;f_Ruminococcaceae;g_Faecalibacterium	ns	CD-G	4.2
	p_Firmicutes;c_Clostridia;o_Clostridiales;f_Veillonellaceae;g_Selenomonas	ns	CD-P	4.1
	p_Firmicutes;c_Clostridia;o_Clostridiales;f_Veillonellaceae;g_Veillonella	63	CD-P	4.0
	p_Fusobacteria;c_Fusobacteriia;o_Fusobacteriales;f_Fusobacteriaceae;g_Fusobacterium	45	CD-G	3.1
	p_Proteobacteria;c_Gammaproteobacteria;o_Enterobacteriales;f_Enterobacteriaceae;g_Escherichia	68	CD-P	4.8
	p_Proteobacteria;c_Gammaproteobacteria;o_Enterobacteriales;f_Enterobacteriaceae;g_Klebsiella	61	CD-P	3.9
Species	p_Actinobacteria;c_Actinobacteria;o_Bifidobacteriales;f_Bifidobacteriaceae;g_Bifidobacterium;s_adolescentis	ns	CD-G	4.3
	p_Actinobacteria;c_Actinobacteria;o_Bifidobacteriales;f_Bifidobacteriaceae;g_Bifidobacterium;s_longum	ns	CD-G	4.3
	p_Actinobacteria;c_Coriobacteriia;o_Coriobacteriales;f_Coriobacteriaceae;g_Collinsella;s_aerofaciens	ns	CD-G	4.3
	p_Bacteroidetes;c_Bacteroidia;o_Bacteroidales;f_Bacteroidaceae;g_Bacteroides;s_plebeius	ns	CD-G	4.0
	p_Bacteroidetes;c_Bacteroidia;o_Bacteroidales;f_Bacteroidaceae;g_Bacteroides;s_uniformis	ns	CD-G	4.0
	p_Firmicutes;c_Bacilli;o_Lactobacillales;f_Streptococcaceae;g_Streptococcus;s_luteciae	ns	CD-P	4.2
	p_Firmicutes;c_Clostridia;o_Clostridiales;f_Veillonellaceae;g_Veillonella;s_dispar	92	CD-P	4.3
	p_Firmicutes;c_Clostridia;o_Clostridiales;f_Lachnospiraceae;g_Blautia;s_producta	ns	CD-G	4.1
	p_Firmicutes;c_Clostridia;o_Clostridiales;f_Ruminococcaceae;g_Faecalibacterium;s_prausnitzii	ns	CD-G	4.5
	p_Proteobacteria;c_Gammaproteobacteria;o_Enterobacteriales;f_Enterobacteriaceae;g_Escherichia;s_coli	96	CD-P	4.9

^a^ number of genus: 314, number of species: 459. ^b^ W by ANCOM analysis. ^c^ The group showing high abundance, by LDA effect size (LEfSe) analysis. ^d^ LDA score by LEfSe analysis. c_ = class; CD-G = good prognosis group; CD-P = poor prognosis group; f_ = family; g_ = genus; LDA = linear discriminant analysis; ns = not significant; o_ = order; p_ = phylum; s_ = specie.

## Supplementary Materials

The following are available online at https://www.mdpi.com/2077-0383/9/6/1748/s1, Table S1: Significantly different taxa between the HC and CD group, Table S2: Comparisons of the alpha diversity between different groups in pairwise Kruskal-Wallis test, Table S3: Beta diversity between groups in pairwise PERMANOVA, Table S4: Predicted MetaCyc pathway abundances significantly associated (based on Welch’s *t*-test [STAMP]) with severity of Crohn’s disease (good vs. poor), Table S5: Predicted enzyme classification (EC) gene families significantly associated (based on Welch’s *t*-test [STAMP]) with severity of Crohn’s disease (good vs. poor), Figure S1: Prediction of the correlation of metagenome functional content with Crohn’s disease prognosis using PICRUSt.
