# Supplementary material for: Differentially Abundant Bacterial Taxa Associated with Prognostic Variables of Crohn’s Disease: Results from the IMPACT Study
Source: J Clin Med. 2020 Jun 5;9(6):1748. doi: 10.3390/jcm9061748 (PMC7357029; doi:10.3390/jcm9061748)
Supplement: Supplementary file 1 [file jcm-09-01748-s001.pdf]

Supplemental Tables

Supplemental Table S1. Significantly different taxa between the HC and CD group

Taxa Level[a]	Taxonomic assignment	W[b]
Phylum	p__Actinobacteria	9
	p__Firmicutes	9
	p__Proteobacteria	9
	p__TM7	9
	p__Verrucomicrobia	9
Class	p__Actinobacteria;c__Actinobacteria	13
	p__Actinobacteria;c__Coriobacteriia	14
	p__Firmicutes;c__Bacilli	14
	p__Firmicutes;c__Clostridia	14
	p__Firmicutes;c__Erysipelotrichi	13
	p__Fusobacteria;c__Fusobacteriia	13
	p__Proteobacteria;c__Alphaproteobacteria	13
	p__Proteobacteria;c__Betaproteobacteria	13
	p__Proteobacteria;c__Deltaproteobacteria	13
	p__Proteobacteria;c__Gammaproteobacteria	13
	p__Verrucomicrobia;c__Verrucomicrobiae	14
orders	p_Actinobacteria;c_Actinobacteria;o_Bifidobacteriales	16
	p_Actinobacteria;c_Coriobacteriia;o_Coriobacteriales	16
	p_Firmicutes;c_Bacilli;o_Lactobacillales	16
	p_Firmicutes;c_Bacilli;o_Turicibacterales	16
	p_Firmicutes;c_Clostridia;o_Clostridiales	14
	p_Fusobacteria;c_Fusobacteriia;o_Fusobacteriales	16
	p_Proteobacteria;c_Betaproteobacteria;o_Burkholderiales	16
	p_Proteobacteria;c_Deltaproteobacteria;o_Desulfovibrionales	16
	p_Proteobacteria;c_Gammaproteobacteria;o_Enterobacteriales	16
families	p_Actinobacteria;c_Actinobacteria;o_Bifidobacteriales;f_Bifidobacteriaceae	34
	p_Actinobacteria;c_Coriobacteriia;o_Coriobacteriales;f_Coriobacteriaceae	33
	p_Bacteroidetes;c_Bacteroidia;o_Bacteroidales;f_Porphyromonadaceae	31
	p_Bacteroidetes;c_Bacteroidia;o_Bacteroidales;f_Prevotellaceae	34
	p_Bacteroidetes;c_Bacteroidia;o_Bacteroidales;f_Rikenellaceae	34
	p_Bacteroidetes;c_Bacteroidia;o_Bacteroidales;f_[Barnesiellaceae]	33
	p_Bacteroidetes;c_Bacteroidia;o_Bacteroidales;f_[Odoribacteraceae]	33
	p_Bacteroidetes;c_Bacteroidia;o_Bacteroidales;f_[Paraprevotellaceae]	35
	p_Firmicutes;c_Bacilli;o_Lactobacillales;f_Carnobacteriaceae	33
	p_Firmicutes;c_Bacilli;o_Lactobacillales;f_Enterococcaceae	35
	p_Firmicutes;c_Bacilli;o_Lactobacillales;f_Lactobacillaceae	33

	p_Firmicutes;c_Bacilli;o_Lactobacillales;f_Streptococcaceae	35
	p_Firmicutes;c_Bacilli;o_Turicibacterales;f_Turicibacteraceae	35
	p_Firmicutes;c_Clostridia;o_Clostridiales;f_Lachnospiraceae	33
	p_Firmicutes;c_Clostridia;o_Clostridiales;f_Peptostreptococcaceae	34
	p_Firmicutes;c_Clostridia;o_Clostridiales;f_Ruminococcaceae	32
	p_Firmicutes;c_Clostridia;o_Clostridiales;f_Veillonellaceae	33
	p_Firmicutes;c_Clostridia;o_Clostridiales;f_[Mogibacteriaceae]	33
	p_Firmicutes;c_Erysipelotrichi;o_Erysipelotrichales;f_Erysipelotrichaceae	33
	p_Fusobacteria;c_Fusobacteriia;o_Fusobacteriales;f_Fusobacteriaceae	33
	p_Proteobacteria;c_Betaproteobacteria;o_Burkholderiales;f_Alcaligenaceae	32
	p_Proteobacteria;c_Gammaproteobacteria;o_Enterobacteriales;f_Enterobacteriaceae	33
genera	p_Actinobacteria;c_Actinobacteria;o_Bifidobacteriales;f_Bifidobacteriaceae;g_Bifidobacterium	66
	p_Actinobacteria;c_Coriobacteriia;o_Coriobacteriales;f_Coriobacteriaceae;g_Collinsella	67
	p_Actinobacteria;c_Coriobacteriia;o_Coriobacteriales;f_Coriobacteriaceae;g_Eggerthella	66
	p_Bacteroidetes;c_Bacteroidia;o_Bacteroidales;f_Porphyromonadaceae;g_Parabacteroides	67
	p_Bacteroidetes;c_Bacteroidia;o_Bacteroidales;f_Prevotellaceae;g_Prevotella	70
	p_Bacteroidetes;c_Bacteroidia;o_Bacteroidales;f_[Odoribacteraceae];g_Butyricimonas	64
	p_Bacteroidetes;c_Bacteroidia;o_Bacteroidales;f_[Odoribacteraceae];g_Odoribacter	67
	p_Firmicutes;c_Bacilli;o_Lactobacillales;f_Carnobacteriaceae;g_Granulicatella	64
	p_Firmicutes;c_Bacilli;o_Lactobacillales;f_Enterococcaceae;g_Enterococcus	68
	p_Firmicutes;c_Bacilli;o_Lactobacillales;f_Lactobacillaceae;g_Lactobacillus	64
	p_Firmicutes;c_Bacilli;o_Lactobacillales;f_Streptococcaceae;g_Streptococcus	70
	p_Firmicutes;c_Clostridia;o_Clostridiales;f_Lachnospiraceae;g_Anaerostipes	64
	p_Firmicutes;c_Clostridia;o_Clostridiales;f_Lachnospiraceae;g_Blautia	66
	p_Firmicutes;c_Clostridia;o_Clostridiales;f_Lachnospiraceae;g_[Ruminococcus]	65
	p_Firmicutes;c_Clostridia;o_Clostridiales;f_Ruminococcaceae;g_Faecalibacterium	70
	p_Proteobacteria;c_Betaproteobacteria;o_Burkholderiales;f_Alcaligenaceae;g_Sutterella	64
	p_Proteobacteria;c_Gammaproteobacteria;o_Enterobacteriales;f_Enterobacteriaceae;g_Escherichia	69
	p_Proteobacteria;c_Gammaproteobacteria;o_Enterobacteriales;f_Enterobacteriaceae;g_Klebsiella	63
Species	p_Actinobacteria;c_Actinobacteria;o_Bifidobacteriales;f_Bifidobacteriaceae;g_Bifidobacterium;s_longum	96
	p_Actinobacteria;c_Coriobacteriia;o_Coriobacteriales;f_Coriobacteriaceae;g_Collinsella;s_aerofaciens	95
	p_Actinobacteria;c_Coriobacteriia;o_Coriobacteriales;f_Coriobacteriaceae;g_Eggerthella;s_lenta	90
	p_Bacteroidetes;c_Bacteroidia;o_Bacteroidales;f_Bacteroidaceae;g_Bacteroides;s_plebeius	97
	p_Bacteroidetes;c_Bacteroidia;o_Bacteroidales;f_Bacteroidaceae;g_Bacteroides;s_uniformis	93
	p_Bacteroidetes;c_Bacteroidia;o_Bacteroidales;f_Prevotellaceae;g_Prevotella;s_copri	97
	p_Firmicutes;c_Clostridia;o_Clostridiales;f_Lachnospiraceae;g_Blautia;s_producta	90
	p_Firmicutes;c_Clostridia;o_Clostridiales;f_Lachnospiraceae;g_[Ruminococcus];s_gnavus	89
	p_Firmicutes;c_Clostridia;o_Clostridiales;f_Ruminococcaceae;g_Faecalibacterium;s_prausnitzii	96
	p_Firmicutes;c_Clostridia;o_Clostridiales;f_Veillonellaceae;g_Veillonella;s_dispar	89

p_Firmicutes;c_Erysipelotrichi;o_Erysipelotrichales;f_Erysipelotrichaceae;g_[Eubacterium];s_dolichum	90
p_Proteobacteria;c_Gammaproteobacteria;o_Enterobacteriales;f_Enterobacteriaceae;g_Escherichia;s_coli	95

c_, class; f_, family; g_, genus; o_, order; p_, phylum; s_, species.

[a] # of phylum: 18, # of class: 39, # of order: 71, # of family: 129, # of genus: 314, # of species: 459.

[b] W=X for taxon k, then H_{0k} is rejected X times. The W statistic for the significantly different taxa relative to more than 90 percent other taxa in each taxa level is shown.

Supplemental Table S2. Comparisons of the alpha diversity between different groups in pairwise Kruskal-Wallis test

Alpha diversity index	Group	Group	H	*P*-value	*q*-value
Observed ASVs[a]	HC (n=623)	CD-G (n=133)	137.333639	1.02E-31	3.16E-31
		CD-I (n=148)	137.270029	1.05E-31	3.16E-31
		CD-P (n=86)	103.174324	3.07E-24	6.14E-24
	CD-G (n=133)	CD-I (n=148)	1.020459	0.31241016	0.37489219
		CD-P (n=86)	0.27932619	0.5971432	0.5971432
	CD-I (n=148)	CD-P (n=86)	1.9326595	0.16446778	0.24670166
Faith's PD[a]	HC (n=623)	CD-G (n=133)	160.611848	8.32E-37	4.98E-36
		CD-I (n=148)	159.240069	1.66E-36	4.98E-36
		CD-P (n=86)	113.113096	2.04E-26	4.08E-26
	CD-G (n=133)	CD-I (n=148)	0.2724429	0.60169775	0.60169775
		CD-P (n=86)	0.70318714	0.40171484	0.4820578
	CD-I (n=148)	CD-P (n=86)	1.72121775	0.18953637	0.28430455
Shannon[a]	HC (n=623)	CD-G (n=133)	143.677726	4.18E-33	8.36E-33
		CD-I (n=148)	172.661523	1.94E-39	1.16E-38
		CD-P (n=86)	151.074029	1.01E-34	3.03E-34
	CD-G (n=133)	CD-I (n=148)	0.0011436	0.97302295	0.97302295
		CD-P (n=86)	12.9673067	0.00031698	0.00038037
	CD-I (n=148)	CD-P (n=86)	14.0443225	0.00017855	0.00026783

CD-G, good prognosis group; CD-I, intermediate prognosis group; CD-P, poor prognosis group; HC, health control.

[a]The p-values among all groups were estimated using Kruskal-Wallis test. The H=286.4, 328.2., and 357.5; p-value=8.5 ×10-62, 7.6 ×10-71, and 3.4 ×10-77 for the observed ASVs, Faith’s PD, and Shannon, respectively.

[*] $q < 0.05$; [**] $q < 0.01$

Supplemental Table S3. Beta diversity between groups in pairwise PERMANOVA

**Beta-diversity index**	**Group**	**Group**	**Sample size**	**Permutations**	**pseudo-F**	***P*-value**	***q*-value**
Unweighted UniFrac distance[a]	HC	CD-G	756	999	70.7970734	0.001	0.0015
		CD-I	771	999	83.1744431	0.001	0.0015
		CD-P	709	999	65.9150438	0.001	0.0015
	CD-G	CD-I	281	999	0.85949129	0.601	0.601
		CD-P	219	999	3.85104199	0.001	0.0015
	CD-I	CD-P	234	999	2.94081876	0.002	0.0024
Weighted UniFrac distance[a]	HC	CD-G	756	999	89.0770595	0.001	0.0015
		CD-I	771	999	98.6220236	0.001	0.0015
		CD-P	709	999	78.2382813	0.001	0.0015
	CD-G	CD-I	281	999	1.00765094	0.393	0.393
		CD-P	219	999	5.81652015	0.001	0.0015
	CD-I	CD-P	234	999	3.57010942	0.004	0.0048

CD-G, good prognosis group; CD-I, intermediate prognosis group; CD-P, poor prognosis group; HC, health control.

[a]The p-values among all groups were estimated using permutational multivariate analysis of variance (PERMANOVA). The psuedo-F=54.9 and 65.9; *P*-value=0.001 and 0.001 for the unweighted UniFrac and weighted UniFrac, respectively.

Supplemental Table S4. Predicted MetaCyc pathway abundances significantly associated (based on Welch's t-test [STAMP]) with severity of Crohn's disease (good vs. poor)

**Metacyc**	**Metacyc_abbreviation**	**Good: mean rel. freq. (%)**	**Good: std. dev. (%)**	**Poor: mean rel. freq. (%)**	**Poor: std. dev. (%)**	**Difference between means**	**95.0% lower CI**	**95.0% upper CI**	**Ratio_ proportions**	***P*-values**	***P*-values (corrected)**
4-hydroxyphenylacetate degradation	3-HYDROXYPHENYLACETATE-DEGRADATION-PWY	0.022	0.046	0.066	0.086	-0.045	-0.064	-0.025	3.072	1.99E-05	7.72.E-03
3-phenylpropanoate and 3-(3-hydroxyphenyl) propanoate degradation to 2-oxopent-4-enoate	HCAMHPDEG-PWY	0.020	0.043	0.059	0.084	-0.040	-0.059	-0.020	2.997	8.91E-05	3.46.E-02
cinnamate and 3-hydroxycinnamate degradation to 2-oxopent-4-enoate	PWY-6690	0.020	0.043	0.059	0.084	-0.040	-0.059	-0.020	2.997	8.91E-05	3.46.E-02
superpathway of phenylethylamine degradation	PWY-6071	0.024	0.050	0.072	0.098	-0.047	-0.070	-0.025	2.950	5.84E-05	2.26.E-02
phenylacetate degradation I (aerobic)	PWY0-321	0.026	0.052	0.074	0.100	-0.049	-0.072	-0.026	2.906	5.67E-05	2.20.E-02
D-glucarate degradation I	GLUCARDEG-PWY	0.034	0.055	0.097	0.114	-0.063	-0.089	-0.037	2.889	4.28E-06	1.66.E-03
ubiquinol-10 biosynthesis (prokaryotic)	PWY-5857	0.032	0.059	0.091	0.098	-0.059	-0.082	-0.036	2.866	1.44E-06	5.57.E-04
ubiquinol-7 biosynthesis (prokaryotic)	PWY-5855	0.032	0.059	0.091	0.098	-0.059	-0.082	-0.036	2.866	1.44E-06	5.57.E-04
ubiquinol-8 biosynthesis (prokaryotic)	PWY-6708	0.032	0.059	0.091	0.098	-0.059	-0.082	-0.036	2.866	1.44E-06	5.57.E-04
ubiquinol-9 biosynthesis (prokaryotic)	PWY-5856	0.032	0.059	0.091	0.098	-0.059	-0.082	-0.036	2.866	1.44E-06	5.57.E-04
superpathway of ubiquinol-8 biosynthesis (prokaryotic)	UBISYN-PWY	0.031	0.059	0.089	0.096	-0.058	-0.081	-0.035	2.854	1.41E-06	5.45.E-04
superpathway of L-arginine, putrescine, and 4-aminobutanoate degradation	ARGDEG-PWY	0.028	0.057	0.080	0.099	-0.051	-0.075	-0.028	2.819	2.46E-05	9.56.E-03
superpathway of L-arginine and L-ornithine degradation	ORNARGDEG-PWY	0.028	0.057	0.080	0.099	-0.051	-0.075	-0.028	2.819	2.46E-05	9.56.E-03
polymyxin resistance	PWY0-1338	0.024	0.051	0.068	0.083	-0.044	-0.064	-0.024	2.818	2.15E-05	8.33.E-03
enterobacterial common antigen biosynthesis	ECASYN-PWY	0.029	0.056	0.083	0.091	-0.053	-0.075	-0.032	2.811	2.60E-06	1.01.E-03
D-galactarate degradation I	GALACTARDEG-PWY	0.032	0.053	0.089	0.097	-0.057	-0.080	-0.035	2.802	1.72E-06	6.66.E-04
superpathway of D-glucarate and D-galactarate degradation	GLUCARGALACTSUPER-PWY	0.032	0.053	0.089	0.097	-0.057	-0.080	-0.035	2.802	1.72E-06	6.66.E-04

superpathway of ornithine degradation	ORNDEG-PWY	0.030	0.062	0.081	0.104	-0.052	-0.076	-0.027	2.746	6.13E-05	2.38.E-02
superpathway of heme biosynthesis from glycine	PWY-5920	0.017	0.031	0.045	0.059	-0.029	-0.042	-0.015	2.741	5.83E-05	2.26.E-02
3-phenylpropanoate and 3-(3-hydroxyphenyl) propanoate degradation	PWY0-1277	0.027	0.053	0.074	0.094	-0.047	-0.069	-0.025	2.734	4.53E-05	1.76.E-02
fatty acid beta oxidation I	FAO-PWY	0.059	0.104	0.160	0.171	-0.101	-0.141	-0.060	2.703	2.49E-06	9.65.E-04
superpathway of glycol metabolism and degradation	GLYCOL-GLYOXDEG-PWY	0.030	0.056	0.079	0.094	-0.049	-0.071	-0.027	2.654	2.01E-05	7.80.E-03
superpathway of glyoxylate bypass and TCA	TCA-GLYOX-BYPASS	0.048	0.083	0.125	0.122	-0.077	-0.107	-0.048	2.611	7.92E-07	3.07.E-04
superpathway of lipopolysaccharide biosynthesis	LPSSYN-PWY	0.031	0.057	0.081	0.087	-0.049	-0.070	-0.028	2.571	7.67E-06	2.97.E-03
superpathway of chorismate metabolism	ALL-CHORISMATE-PWY	0.050	0.079	0.128	0.114	-0.078	-0.106	-0.050	2.564	1.18E-07	4.56.E-05
enterobactin biosynthesis	ENTBACSYN-PWY	0.052	0.081	0.131	0.117	-0.079	-0.108	-0.051	2.531	1.74E-07	6.73.E-05
ppGpp biosynthesis	PPGPPMET-PWY	0.050	0.085	0.126	0.129	-0.076	-0.107	-0.045	2.516	3.61E-06	1.40.E-03
superpathway of L-threonine metabolism	THREOCAT-PWY	0.031	0.055	0.078	0.086	-0.047	-0.068	-0.026	2.492	1.36E-05	5.27.E-03
glyoxylate cycle	GLYOXYLATE-BYPASS	0.042	0.072	0.104	0.101	-0.062	-0.087	-0.037	2.491	2.10E-06	8.14.E-04
superpathway of glycolysis, pyruvate dehydrogenase, TCA, and glyoxylate bypass	GLYCOLYSIS-TCA-GLYOX-BYPASS	0.071	0.109	0.175	0.143	-0.104	-0.140	-0.069	2.461	4.16E-08	1.61.E-05
heme biosynthesis I (aerobic)	HEME-BIOSYNTHESIS-II	0.046	0.072	0.112	0.106	-0.066	-0.092	-0.041	2.440	9.56E-07	3.71.E-04
superpathway of (Kdo)2-lipid A biosynthesis	KDO-NAGLIPASYN-PWY	0.051	0.076	0.122	0.104	-0.071	-0.097	-0.045	2.379	1.91E-07	7.41.E-05
TCA cycle IV (2-oxoglutarate decarboxylase)	P105-PWY	0.076	0.109	0.179	0.136	-0.103	-0.138	-0.069	2.363	1.93E-08	7.48.E-06
toluene degradation I (aerobic) (via o-cresol)	PWY-5180	0.032	0.054	0.076	0.088	-0.044	-0.064	-0.023	2.353	5.97E-05	2.32.E-02
toluene degradation II (aerobic) (via 4-methylcatechol)	PWY-5182	0.032	0.054	0.076	0.088	-0.044	-0.064	-0.023	2.353	5.97E-05	2.32.E-02
superpathway of demethylmenaquinol-6 biosynthesis I	PWY-5860	0.050	0.068	0.117	0.099	-0.067	-0.091	-0.043	2.325	1.83E-07	7.11.E-05
superpathway of demethylmenaquinol-9 biosynthesis	PWY-5862	0.050	0.068	0.117	0.099	-0.067	-0.091	-0.043	2.325	1.84E-07	7.15.E-05

1,4-dihydroxy-2-naphthoate biosynthesis I	PWY-5837	0.055	0.079	0.129	0.114	-0.073	-0.101	-0.046	2.323	5.38E-07	2.09.E-04
superpathway of L-tryptophan biosynthesis	PWY-6629	0.078	0.118	0.178	0.142	-0.100	-0.136	-0.063	2.275	1.97E-07	7.65.E-05
methylphosphonate degradation I	PWY0-1533	0.041	0.057	0.092	0.101	-0.052	-0.075	-0.028	2.274	2.98E-05	1.16.E-02
superpathway of heme biosynthesis from uroporphyrinogen-III	PWY0-1415	0.044	0.062	0.100	0.097	-0.056	-0.079	-0.033	2.263	4.46E-06	1.73.E-03
superpathway of phylloquinol biosynthesis	PWY-5863	0.060	0.082	0.136	0.114	-0.076	-0.104	-0.048	2.257	3.36E-07	1.31.E-04
superpathway of menaquinol-10 biosynthesis	PWY-5896	0.058	0.074	0.131	0.102	-0.073	-0.098	-0.048	2.253	4.97E-08	1.93.E-05
superpathway of menaquinol-6 biosynthesis I	PWY-5850	0.058	0.074	0.131	0.102	-0.073	-0.098	-0.048	2.253	4.97E-08	1.93.E-05
superpathway of menaquinol-9 biosynthesis	PWY-5845	0.058	0.074	0.131	0.102	-0.073	-0.098	-0.048	2.252	5.00E-08	1.94.E-05
superpathway of demethylmenaquinol-8 biosynthesis	PWY-5861	0.072	0.090	0.156	0.118	-0.083	-0.113	-0.054	2.149	1.02E-07	3.96.E-05
superpathway of menaquinol-8 biosynthesis I	PWY-5838	0.081	0.092	0.168	0.120	-0.088	-0.118	-0.058	2.088	3.98E-08	1.55.E-05
superpathway of menaquinol-11 biosynthesis	PWY-5897	0.080	0.091	0.166	0.119	-0.086	-0.115	-0.056	2.067	6.20E-08	2.41.E-05
superpathway of menaquinol-12 biosynthesis	PWY-5898	0.080	0.091	0.166	0.119	-0.086	-0.115	-0.056	2.067	6.20E-08	2.41.E-05
superpathway of menaquinol-13 biosynthesis	PWY-5899	0.080	0.091	0.166	0.119	-0.086	-0.115	-0.056	2.067	6.20E-08	2.41.E-05
superpathay of heme biosynthesis from glutamate	PWY-5918	0.071	0.089	0.146	0.113	-0.074	-0.103	-0.046	2.045	6.67E-07	2.59.E-04
superpathway of menaquinol-7 biosynthesis	PWY-5840	0.084	0.092	0.169	0.117	-0.085	-0.114	-0.055	2.009	5.57E-08	2.16.E-05
tRNA processing	PWY0-1479	0.075	0.078	0.147	0.102	-0.072	-0.097	-0.046	1.962	1.02E-07	3.94.E-05
ADP-L-glycero-β-D-manno-heptose biosynthesis	PWY0-1241	0.066	0.083	0.128	0.109	-0.062	-0.089	-0.035	1.939	1.27E-05	4.93.E-03
superpathway of L-methionine biosynthesis (by sulfhydrylation)	PWY-5345	0.087	0.099	0.163	0.106	-0.076	-0.104	-0.048	1.869	3.28E-07	1.27.E-04
(5Z)-dodec-5-enoate biosynthesis	PWY0-862	0.223	0.195	0.359	0.204	-0.137	-0.191	-0.082	1.613	1.81E-06	7.03.E-04
superpathway of histidine, purine, and pyrimidine biosynthesis	PRPP-PWY	0.222	0.157	0.349	0.142	-0.127	-0.167	-0.087	1.572	3.01E-09	1.17.E-06
anhydromuropeptides recycling	PWY0-1261	0.199	0.159	0.310	0.182	-0.111	-0.158	-0.064	1.558	6.38E-06	2.47.E-03

Calvin-Benson-Bassham cycle	CALVIN-PWY	0.860	0.136	0.778	0.151	0.081	0.042	0.121	0.905	7.36E-05	2.86.E-02
tRNA charging	TRNA-CHARGING-PWY	0.735	0.118	0.662	0.125	0.074	0.040	0.107	0.900	2.11E-05	8.21.E-03
superpathway of aromatic amino acid biosynthesis	COMPLETE-ARO-PWY	0.790	0.142	0.707	0.139	0.082	0.044	0.120	0.896	3.12E-05	1.21.E-02
glycolysis III (from glucose)	ANAGLYCOLYSIS-PWY	0.899	0.136	0.805	0.146	0.095	0.056	0.133	0.895	2.94E-06	1.14.E-03
5-aminoimidazole ribonucleotide biosynthesis I	PWY-6121	0.822	0.121	0.735	0.146	0.087	0.050	0.124	0.894	8.41E-06	3.26.E-03
superpathway of L-isoleucine biosynthesis I	PWY-3001	0.766	0.129	0.684	0.129	0.082	0.047	0.117	0.893	7.56E-06	2.94.E-03
chorismate biosynthesis I	ARO-PWY	0.761	0.137	0.679	0.135	0.082	0.045	0.119	0.893	2.20E-05	8.53.E-03
superpathway of branched amino acid biosynthesis	BRANCHED-CHAIN-AA-SYN-PWY	0.785	0.151	0.697	0.154	0.088	0.047	0.130	0.888	4.40E-05	1.71.E-02
L-isoleucine biosynthesis I (from threonine)	ILEUSYN-PWY	0.855	0.161	0.758	0.165	0.098	0.053	0.142	0.886	2.44E-05	9.48.E-03
L-valine biosynthesis	VALSYN-PWY	0.855	0.161	0.758	0.165	0.098	0.053	0.142	0.886	2.44E-05	9.48.E-03
L-isoleucine biosynthesis III	PWY-5103	0.770	0.154	0.682	0.161	0.088	0.045	0.131	0.886	8.08E-05	3.14.E-02
L-isoleucine biosynthesis II	PWY-5101	0.899	0.171	0.795	0.175	0.104	0.056	0.151	0.885	2.39E-05	9.26.E-03
pentose phosphate pathway (non-oxidative branch)	NONOXIPENT-PWY	1.135	0.188	1.000	0.202	0.136	0.082	0.189	0.880	1.31E-06	5.10.E-04
L-lysine biosynthesis VI	PWY-5097	0.804	0.132	0.702	0.161	0.102	0.061	0.143	0.874	2.20E-06	8.53.E-04
5-aminoimidazole ribonucleotide biosynthesis II	PWY-6122	0.855	0.155	0.741	0.174	0.114	0.068	0.159	0.867	1.83E-06	7.12.E-04
superpathway of 5-aminoimidazole ribonucleotide biosynthesis	PWY-6277	0.855	0.155	0.741	0.174	0.114	0.068	0.159	0.867	1.83E-06	7.12.E-04
superpathway of L-serine and glycine biosynthesis I	SER-GLYSYN-PWY	0.707	0.156	0.611	0.170	0.095	0.051	0.140	0.865	4.21E-05	1.63.E-02
L-lysine biosynthesis I	DAPLYSINESYN-PWY	0.636	0.160	0.547	0.143	0.089	0.048	0.130	0.860	2.71E-05	1.05.E-02
pyruvate fermentation to isobutanol (engineered)	PWY-7111	1.080	0.276	0.928	0.249	0.152	0.081	0.222	0.860	3.65E-05	1.42.E-02
NAD salvage pathway I	PYRIDNUCSAL-PWY	0.547	0.111	0.470	0.155	0.077	0.039	0.115	0.860	1.00E-04	3.88.E-02
adenine and adenosine salvage III	PWY-6609	0.746	0.200	0.636	0.199	0.110	0.056	0.164	0.852	9.01E-05	3.50.E-02
starch degradation V	PWY-6737	0.899	0.178	0.764	0.226	0.135	0.078	0.191	0.850	6.02E-06	2.34.E-03
superpathway of geranylgeranyl diphosphate biosynthesis II (via MEP)	PWY-5121	0.673	0.128	0.565	0.159	0.108	0.068	0.148	0.840	3.53E-07	1.37.E-04
methylerythritol phosphate pathway I	NONMEVIPP-PWY	0.677	0.138	0.565	0.174	0.112	0.068	0.156	0.835	1.14E-06	4.42.E-04
methylerythritol phosphate pathway II	PWY-7560	0.677	0.138	0.565	0.174	0.112	0.068	0.156	0.835	1.14E-06	4.42.E-04

glycogen degradation I (bacterial)	GLYCOCAT-PWY	0.851	0.185	0.705	0.219	0.146	0.090	0.202	0.829	7.85E-07	3.04.E-04
L-isoleucine biosynthesis IV	PWY-5104	0.858	0.188	0.704	0.188	0.154	0.103	0.205	0.821	1.43E-08	5.54.E-06
adenosylcobalamin salvage from cobinamide I	COBALSYN-PWY	0.545	0.187	0.442	0.176	0.104	0.055	0.153	0.810	4.55E-05	1.77.E-02
glycogen biosynthesis I (from ADP-D-Glucose)	GLYCOGENSYNTH-PWY	0.809	0.233	0.649	0.243	0.160	0.095	0.225	0.802	2.41E-06	9.37.E-04
adenosylcobalamin salvage from cobinamide II	PWY-6269	0.519	0.179	0.401	0.168	0.118	0.071	0.165	0.773	1.50E-06	5.82.E-04
adenosylcobalamin biosynthesis from cobyrinate a,c-diamide I	PWY-5509	0.515	0.178	0.397	0.167	0.118	0.072	0.165	0.770	1.21E-06	4.68.E-04
thiazole biosynthesis I (E. coli)	PWY-6892	0.571	0.148	0.434	0.176	0.137	0.092	0.182	0.760	1.17E-08	4.54.E-06
L-glutamate and L-glutamine biosynthesis	PWY-5505	0.550	0.225	0.408	0.212	0.143	0.084	0.202	0.741	3.66E-06	1.42.E-03
pyrimidine deoxyribonucleotides biosynthesis from CTP	PWY-7210	0.137	0.086	0.085	0.088	0.052	0.028	0.076	0.621	2.49E-05	9.66.E-03
pyrimidine deoxyribonucleotides de novo biosynthesis IV	PWY-7198	0.114	0.076	0.070	0.077	0.044	0.023	0.065	0.615	5.05E-05	1.96.E-02
methanogenesis from acetate	METH-ACETATE-PWY	0.329	0.224	0.186	0.202	0.143	0.086	0.200	0.566	1.83E-06	7.12.E-04

Supplemental Table S5. Predicted enzyme classification (EC) gene families significantly associated (based on Welch's t-test [STAMP]) with severity of Crohn's disease (good vs. poor)

EC_level4	EC number	Good: mean rel. freq. (%)	Good: std. dev. (%)	Poor: mean rel. freq. (%)	Poor: std. dev. (%)	Difference between means	95.0% lower CI	95.0% upper CI	Ratio of mean proportion	*P*-values	*P*-values (corrected)
Flavin reductase (NADH)	EC:1.5.1.36	0.004	0.010	0.014	0.019	-0.010	-0.014	-0.006	3.274	1.46E-05	2.89.E-02
Glyoxylate reductase (NADP(+))	EC:1.1.1.79	0.006	0.012	0.019	0.024	-0.013	-0.019	-0.007	3.260	9.20E-06	1.82.E-02
Thymidine phosphorylase	EC:2.4.2.4	0.005	0.010	0.015	0.019	-0.010	-0.015	-0.006	3.227	8.21E-06	1.63.E-02
D-malate dehydrogenase (decarboxylating)	EC:1.1.1.83	0.006	0.011	0.018	0.024	-0.012	-0.018	-0.007	3.202	2.35E-05	4.66.E-02
Tartrate decarboxylase	EC:4.1.1.73	0.006	0.011	0.018	0.024	-0.012	-0.018	-0.007	3.202	2.35E-05	4.66.E-02
Tartrate dehydrogenase	EC:1.1.1.93	0.006	0.011	0.018	0.024	-0.012	-0.018	-0.007	3.202	2.35E-05	4.66.E-02
Phosphoribulokinase	EC:2.7.1.19	0.005	0.010	0.014	0.019	-0.010	-0.014	-0.006	3.173	1.56E-05	3.10.E-02
dTDP-4-amino-4,6-dideoxy-D-galactose acyltransferase	EC:2.3.1.210	0.004	0.010	0.014	0.019	-0.010	-0.014	-0.005	3.169	1.67E-05	3.31.E-02
Aquacobalamin reductase	EC:1.16.1.3	0.004	0.010	0.014	0.019	-0.010	-0.014	-0.005	3.169	1.67E-05	3.31.E-02
Inosine kinase	EC:2.7.1.73	0.004	0.010	0.014	0.019	-0.010	-0.014	-0.005	3.169	1.67E-05	3.31.E-02
Riboflavin reductase (NAD(P)H)	EC:1.5.1.41	0.004	0.010	0.014	0.019	-0.010	-0.014	-0.005	3.169	1.67E-05	3.31.E-02
TDP-N-acetylfucosamine:lipid II N-acetylfucosaminyltransferase	EC:2.4.1.325	0.004	0.010	0.014	0.019	-0.010	-0.014	-0.005	3.169	1.67E-05	3.31.E-02
Thiamine kinase	EC:2.7.1.89	0.004	0.010	0.014	0.019	-0.010	-0.014	-0.005	3.169	1.67E-05	3.31.E-02
Vitamin B12-transporting ATPase	EC:3.6.3.33	0.004	0.010	0.014	0.019	-0.010	-0.014	-0.005	3.169	1.67E-05	3.31.E-02
Kdo(2)-lipid IV(A) palmitoleoyltransferase	EC:2.3.1.242	0.004	0.010	0.014	0.019	-0.010	-0.014	-0.005	3.169	1.67E-05	3.32.E-02
UDP-4-amino-4-deoxy-L-arabinose aminotransferase	EC:2.6.1.87	0.005	0.010	0.014	0.019	-0.010	-0.014	-0.005	3.160	1.72E-05	3.40.E-02
Deoxyribonuclease V	EC:3.1.21.7	0.005	0.010	0.014	0.019	-0.010	-0.014	-0.005	3.152	1.68E-05	3.33.E-02
UDP-sugar diphosphatase	EC:3.6.1.45	0.005	0.009	0.014	0.019	-0.010	-0.014	-0.005	3.151	1.53E-05	3.04.E-02
tRNA(Met) cytidine acetyltransferase	EC:2.3.1.193	0.005	0.009	0.014	0.019	-0.010	-0.014	-0.005	3.148	1.55E-05	3.06.E-02
L-lactate dehydrogenase (cytochrome)	EC:1.1.2.3	0.006	0.012	0.019	0.025	-0.013	-0.018	-0.007	3.137	1.95E-05	3.86.E-02
Molybdopterin adenylyltransferase	EC:2.7.7.75	0.005	0.010	0.015	0.018	-0.010	-0.014	-0.006	3.114	8.25E-06	1.64.E-02
3-hydroxybutyryl-CoA epimerase	EC:5.1.2.3	0.009	0.020	0.029	0.037	-0.020	-0.028	-0.011	3.099	1.40E-05	2.78.E-02
[Acyl-carrier-protein] phosphodiesterase	EC:3.1.4.14	0.005	0.011	0.014	0.019	-0.010	-0.014	-0.005	3.094	1.88E-05	3.73.E-02
Pitrilysin	EC:3.4.24.55	0.005	0.011	0.015	0.019	-0.010	-0.014	-0.005	3.089	1.89E-05	3.74.E-02
Ribosylnicotinamide kinase	EC:2.7.1.22	0.005	0.010	0.015	0.019	-0.010	-0.014	-0.006	3.086	1.32E-05	2.62.E-02
Glutathionylspermidine amidase	EC:3.5.1.78	0.005	0.010	0.014	0.019	-0.010	-0.014	-0.005	3.077	1.61E-05	3.20.E-02
Glutathionylspermidine synthase	EC:6.3.1.8	0.005	0.010	0.014	0.019	-0.010	-0.014	-0.005	3.077	1.61E-05	3.20.E-02
23S rRNA (uracil(747)-C(5))-methyltransferase	EC:2.1.1.189	0.005	0.010	0.015	0.019	-0.010	-0.014	-0.006	3.075	1.29E-05	2.55.E-02
Lauroyl-Kdo(2)-lipid IV(A) myristoyltransferase	EC:2.3.1.243	0.005	0.010	0.014	0.019	-0.010	-0.014	-0.005	3.075	1.62E-05	3.21.E-02
PepB aminopeptidase	EC:3.4.11.23	0.005	0.010	0.014	0.019	-0.010	-0.014	-0.005	3.075	1.62E-05	3.21.E-02
Choline dehydrogenase	EC:1.1.99.1	0.005	0.011	0.015	0.019	-0.010	-0.014	-0.005	3.069	2.35E-05	4.65.E-02

Diacylglycerol diphosphate phosphatase	EC:3.1.3.81	0.005	0.010	0.014	0.019	-0.010	-0.014	-0.005	3.069	1.54E-05	3.06.E-02
2-polyprenyl-6-hydroxyphenol methylase	EC:2.1.1.222	0.005	0.011	0.015	0.018	-0.010	-0.015	-0.006	3.068	7.19E-06	1.43.E-02
3-demethylubiquinol 3-O-methyltransferase	EC:2.1.1.64	0.005	0.011	0.015	0.018	-0.010	-0.015	-0.006	3.068	7.19E-06	1.43.E-02
50S ribosomal protein L16 3-hydroxylase	EC:1.14.11.47	0.006	0.013	0.019	0.024	-0.013	-0.018	-0.007	3.061	2.08E-05	4.12.E-02
Phosphatidate phosphatase	EC:3.1.3.4	0.005	0.010	0.015	0.019	-0.010	-0.014	-0.005	3.059	1.55E-05	3.08.E-02
Lipid IV(A) 4-amino-4-deoxy-L-arabinosyltransferase	EC:2.4.2.43	0.005	0.012	0.016	0.020	-0.011	-0.015	-0.006	3.053	2.41E-05	4.77.E-02
[Isocitrate dehydrogenase (NADP(+))] kinase	EC:2.7.11.5	0.005	0.010	0.014	0.019	-0.010	-0.014	-0.005	3.041	2.31E-05	4.59.E-02
HycI peptidase	EC:3.4.23.51	0.005	0.010	0.014	0.019	-0.010	-0.014	-0.005	3.039	1.78E-05	3.53.E-02
Glutathione synthase	EC:6.3.2.3	0.005	0.011	0.015	0.019	-0.010	-0.014	-0.006	3.035	1.16E-05	2.30.E-02
Coproporphyrinogen oxidase	EC:1.3.3.3	0.005	0.011	0.015	0.019	-0.010	-0.014	-0.006	3.035	1.33E-05	2.63.E-02
Phosphogluconate dehydratase	EC:4.2.1.12	0.005	0.011	0.014	0.019	-0.010	-0.014	-0.005	3.005	2.37E-05	4.69.E-02
Isocitrate lyase	EC:4.1.3.1	0.005	0.011	0.014	0.019	-0.010	-0.014	-0.005	3.001	2.31E-05	4.57.E-02
Acetyl-CoA C-acyltransferase	EC:2.3.1.16	0.011	0.022	0.033	0.042	-0.022	-0.032	-0.012	2.984	1.38E-05	2.73.E-02
Deoxyribodipyrimidine photo-lyase	EC:4.1.99.3	0.005	0.011	0.015	0.019	-0.010	-0.015	-0.006	2.975	1.23E-05	2.45.E-02
2-methylisocitrate dehydratase	EC:4.2.1.99	0.005	0.011	0.015	0.019	-0.010	-0.014	-0.006	2.971	1.55E-05	3.08.E-02
Chorismate lyase	EC:4.1.3.40	0.005	0.011	0.015	0.019	-0.010	-0.014	-0.005	2.959	1.89E-05	3.75.E-02
NADPH:quinone reductase	EC:1.6.5.5	0.005	0.011	0.016	0.019	-0.011	-0.015	-0.006	2.948	4.46E-06	8.84.E-03
N-acetylglucosamine kinase	EC:2.7.1.59	0.005	0.011	0.015	0.019	-0.010	-0.014	-0.005	2.945	1.96E-05	3.88.E-02
Tellurite methyltransferase	EC:2.1.1.265	0.008	0.014	0.025	0.032	-0.016	-0.024	-0.009	2.943	1.25E-05	2.48.E-02
3-hydroxy acid dehydrogenase	EC:1.1.1.381	0.005	0.010	0.015	0.019	-0.010	-0.014	-0.006	2.939	8.67E-06	1.72.E-02
Acyl-[acyl-carrier-protein]--phospholipid O-acyltransferase	EC:2.3.1.40	0.005	0.011	0.016	0.020	-0.010	-0.015	-0.006	2.932	1.66E-05	3.30.E-02
tRNA (5-methylaminomethyl-2-thiouridylate)-methyltransferase	EC:2.1.1.61	0.005	0.011	0.016	0.019	-0.010	-0.015	-0.006	2.922	6.63E-06	1.31.E-02
Assimilatory sulfite reductase (NADPH)	EC:1.8.1.2	0.010	0.021	0.030	0.037	-0.020	-0.029	-0.011	2.918	1.05E-05	2.08.E-02
Trans-2-decenoyl-[acyl-carrier-protein] isomerase	EC:5.3.3.14	0.011	0.018	0.032	0.036	-0.021	-0.029	-0.013	2.904	1.53E-06	3.04.E-03
tRNA pseudouridine(65) synthase	EC:5.4.99.26	0.005	0.010	0.016	0.019	-0.010	-0.015	-0.006	2.898	8.25E-06	1.64.E-02
1,4-dihydroxy-2-naphthoyl-CoA hydrolase	EC:3.1.2.28	0.005	0.010	0.015	0.018	-0.010	-0.014	-0.005	2.886	2.26E-05	4.48.E-02
3-hydroxyacyl-CoA dehydrogenase	EC:1.1.1.35	0.011	0.021	0.031	0.037	-0.020	-0.029	-0.011	2.882	9.74E-06	1.93.E-02
Long-chain-fatty-acid--[acyl-carrier-protein] ligase	EC:6.2.1.20	0.005	0.011	0.016	0.019	-0.010	-0.015	-0.006	2.870	1.91E-05	3.78.E-02
Galactarate dehydratase	EC:4.2.1.42	0.006	0.011	0.017	0.019	-0.011	-0.015	-0.006	2.868	3.88E-06	7.70.E-03
Exoribonuclease II	EC:3.1.13.1	0.006	0.010	0.016	0.019	-0.011	-0.015	-0.006	2.853	3.04E-06	6.03.E-03
23S rRNA (cytidine(2498)-2'-O)-methyltransferase	EC:2.1.1.186	0.005	0.011	0.015	0.018	-0.010	-0.014	-0.005	2.848	2.04E-05	4.05.E-02
ATP diphosphatase	EC:3.6.1.8	0.005	0.011	0.015	0.018	-0.010	-0.014	-0.006	2.830	1.16E-05	2.30.E-02
2-dehydro-3-deoxyglucarate aldolase	EC:4.1.2.20	0.007	0.011	0.020	0.022	-0.013	-0.018	-0.008	2.828	1.72E-06	3.42.E-03
Protein-L-isoaspartate(D-aspartate) O-methyltransferase	EC:2.1.1.77	0.006	0.011	0.016	0.019	-0.010	-0.015	-0.006	2.827	8.57E-06	1.70.E-02

3-dehydro-L-gulonate-6-phosphate decarboxylase	EC:4.1.1.85	0.012	0.023	0.033	0.041	-0.022	-0.031	-0.012	2.820	1.71E-05	3.38.E-02
Oxoglutarate dehydrogenase (succinyl-transferring)	EC:1.2.4.2	0.006	0.011	0.016	0.019	-0.011	-0.015	-0.006	2.807	5.13E-06	1.02.E-02
Glucarate dehydratase	EC:4.2.1.40	0.007	0.012	0.019	0.023	-0.012	-0.017	-0.007	2.792	8.27E-06	1.64.E-02
Ribosomal protein L3 N(5)-glutamine methyltransferase	EC:2.1.1.298	0.006	0.011	0.016	0.019	-0.010	-0.015	-0.006	2.784	9.13E-06	1.81.E-02
tRNA (uracil(54)-C(5))-methyltransferase	EC:2.1.1.35	0.006	0.011	0.017	0.020	-0.011	-0.015	-0.006	2.777	2.24E-05	4.45.E-02
Hydroxyacylglutathione hydrolase	EC:3.1.2.6	0.006	0.011	0.016	0.019	-0.010	-0.015	-0.006	2.769	2.29E-05	4.55.E-02
Exodeoxyribonuclease I	EC:3.1.11.1	0.006	0.011	0.016	0.019	-0.010	-0.014	-0.006	2.752	1.72E-05	3.41.E-02
Molybdopterin synthase	EC:2.8.1.12	0.006	0.011	0.017	0.019	-0.011	-0.015	-0.006	2.747	4.42E-06	8.76.E-03
23S rRNA (uridine(2552)-2'-O)-methyltransferase	EC:2.1.1.166	0.006	0.011	0.017	0.019	-0.010	-0.015	-0.006	2.738	4.72E-06	9.36.E-03
Bis(5'-nucleosyl)-tetraphosphatase (symmetrical)	EC:3.6.1.41	0.006	0.011	0.016	0.019	-0.010	-0.014	-0.006	2.714	9.33E-06	1.85.E-02
Deoxyribonuclease I	EC:3.1.21.1	0.006	0.011	0.016	0.019	-0.010	-0.014	-0.006	2.714	1.54E-05	3.06.E-02
Oligopeptidase A	EC:3.4.24.70	0.006	0.011	0.016	0.019	-0.010	-0.014	-0.006	2.668	1.18E-05	2.34.E-02
Lipopolysaccharide N-acetylmannosaminouronosyltransferase	EC:2.4.1.180	0.006	0.011	0.015	0.018	-0.010	-0.014	-0.005	2.653	2.13E-05	4.23.E-02
Galactonate dehydratase	EC:4.2.1.6	0.007	0.011	0.018	0.019	-0.011	-0.016	-0.007	2.639	3.38E-06	6.70.E-03
Adenylate cyclase	EC:4.6.1.1	0.007	0.012	0.019	0.021	-0.012	-0.017	-0.007	2.626	9.29E-06	1.84.E-02
Succinate-semialdehyde dehydrogenase (NAD(P)(+))	EC:1.2.1.16	0.019	0.031	0.050	0.051	-0.031	-0.043	-0.018	2.599	1.86E-06	3.68.E-03
2-succinyl-6-hydroxy-2,4-cyclohexadiene-1-carboxylate synthase	EC:4.2.99.20	0.009	0.015	0.023	0.024	-0.014	-0.020	-0.008	2.564	3.50E-06	6.95.E-03
Phosphonate-transporting ATPase	EC:3.6.3.28	0.010	0.015	0.026	0.027	-0.016	-0.022	-0.010	2.563	2.11E-06	4.17.E-03
Gamma-glutamyl hercynylcysteine S-oxide hydrolase	EC:3.5.1.118	0.006	0.011	0.016	0.018	-0.010	-0.014	-0.005	2.554	1.29E-05	2.55.E-02
ADP-glyceromanno-heptose 6-epimerase	EC:5.1.3.20	0.010	0.017	0.026	0.025	-0.016	-0.022	-0.010	2.522	7.78E-07	1.54.E-03
4-hydroxy-2-oxoheptanedioate aldolase	EC:4.1.2.52	0.009	0.011	0.022	0.023	-0.013	-0.019	-0.008	2.521	1.89E-06	3.75.E-03
Glutarate-semialdehyde dehydrogenase	EC:1.2.1.20	0.015	0.025	0.037	0.038	-0.022	-0.031	-0.013	2.493	5.43E-06	1.08.E-02
Succinate-semialdehyde dehydrogenase (NADP(+))	EC:1.2.1.79	0.015	0.025	0.037	0.038	-0.022	-0.031	-0.013	2.493	5.43E-06	1.08.E-02
tRNA (guanosine(18)-2'-O)-methyltransferase	EC:2.1.1.34	0.007	0.012	0.017	0.018	-0.010	-0.014	-0.006	2.462	1.42E-05	2.82.E-02
o-succinylbenzoate synthase	EC:4.2.1.113	0.010	0.016	0.023	0.024	-0.014	-0.020	-0.008	2.450	5.29E-06	1.05.E-02
Ferrochelatase	EC:4.99.1.1	0.012	0.019	0.030	0.029	-0.017	-0.024	-0.010	2.408	2.05E-06	4.06.E-03
Anhydro-N-acetylmuramic acid kinase	EC:2.7.1.170	0.007	0.011	0.017	0.019	-0.010	-0.014	-0.005	2.320	1.94E-05	3.85.E-02
GMP reductase	EC:1.7.1.7	0.020	0.028	0.046	0.043	-0.026	-0.036	-0.015	2.276	2.67E-06	5.29.E-03
Nitrate reductase	EC:1.7.99.4	0.062	0.079	0.138	0.131	-0.076	-0.107	-0.045	2.212	3.79E-06	7.51.E-03
Phosphatidylglycerophosphatase	EC:3.1.3.27	0.024	0.032	0.053	0.053	-0.029	-0.041	-0.016	2.195	1.23E-05	2.44.E-02
D-glycero-beta-D-manno-heptose 1-phosphate adenylyltransferase	EC:2.7.7.70	0.012	0.017	0.025	0.024	-0.014	-0.019	-0.008	2.156	9.16E-06	1.82.E-02

D-glycero-beta-D-manno-heptose-7-phosphate kinase	EC:2.7.1.167	0.012	0.017	0.025	0.024	-0.014	-0.019	-0.008	2.156	9.16E-06	1.82.E-02
Formate dehydrogenase	EC:1.2.1.2	0.038	0.045	0.082	0.079	-0.044	-0.062	-0.026	2.146	5.88E-06	1.17.E-02
23S rRNA (adenine(2030)-N(6))-methyltransferase	EC:2.1.1.266	0.009	0.013	0.019	0.018	-0.010	-0.015	-0.006	2.145	1.97E-05	3.92.E-02
Pyruvate dehydrogenase (quinone)	EC:1.2.5.1	0.014	0.018	0.030	0.026	-0.016	-0.022	-0.010	2.144	1.81E-06	3.58.E-03
Glycerol-3-phosphate-transporting ATPase	EC:3.6.3.20	0.014	0.014	0.029	0.029	-0.015	-0.021	-0.008	2.065	1.81E-05	3.59.E-02
Glucan 1,3-beta-glucosidase	EC:3.2.1.58	0.013	0.012	0.006	0.009	0.007	0.004	0.010	0.478	2.90E-06	5.76.E-03
Scyllo-inositol 2-dehydrogenase (NAD(+))	EC:1.1.1.370	0.016	0.016	0.007	0.012	0.009	0.005	0.013	0.427	6.06E-06	1.20.E-02

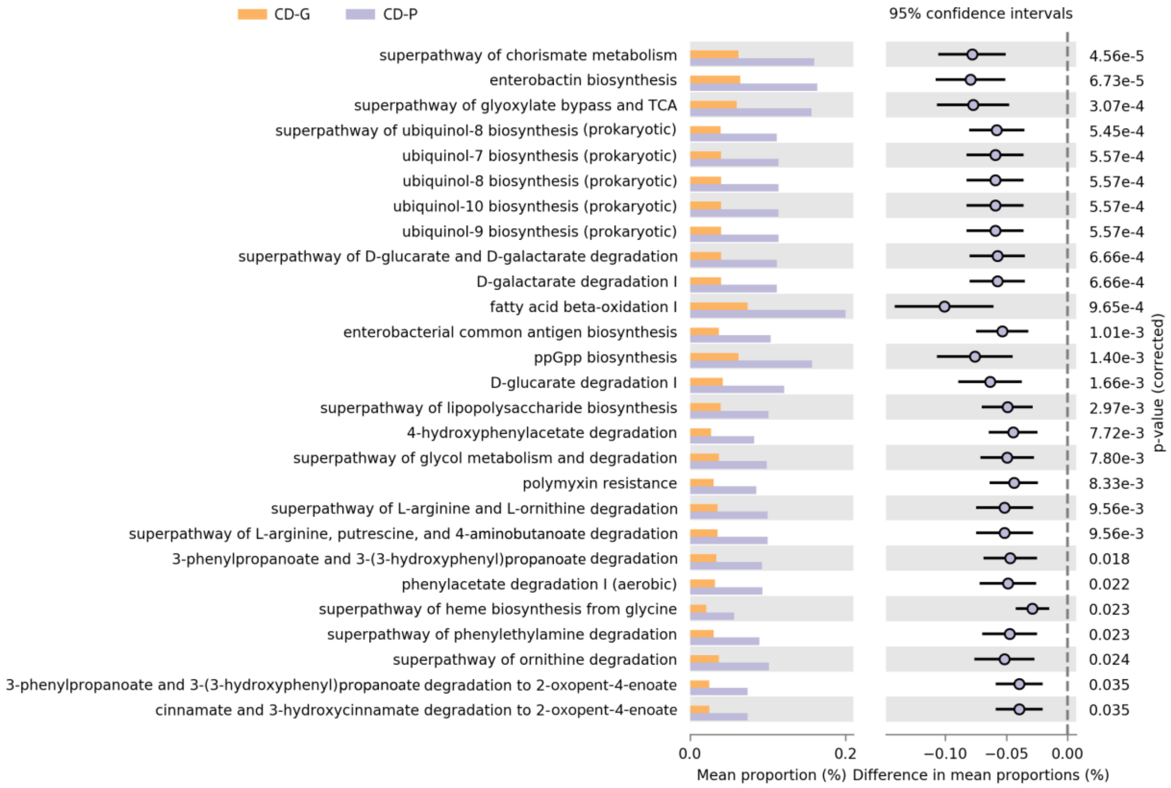

Supplemental Figure S1. Prediction of the correlation of metagenome functional content with Crohn's disease prognosis using PICRUSt. CD-G, good prognosis group; CD-P, poor prognosis group
